# Ziziphi Spinosae Semen Flavonoid Ameliorates Hypothalamic Metabolism and Modulates Gut Microbiota in Chronic Restraint Stress-Induced Anxiety-like Behavior in Mice

**DOI:** 10.3390/foods14050828

**Published:** 2025-02-27

**Authors:** Yan Yan, Ni Zhao, Jiaying Liu, Shengmei Zhang, Yinjie Zhang, Xuemei Qin, Kefeng Zhai, Chenhui Du

**Affiliations:** 1 Modern Research Center for Traditional Chinese Medicine, Shanxi University, Taiyuan 030006, China; yanyan520@sxu.edu.cn (Y.Y.); zhaoni15994300031@163.com (N.Z.); 13834684352@163.com (J.L.); zhyj@sxu.edu.cn (Y.Z.); qinxm@sxu.edu.cn (X.Q.); 2 School of Traditional Chinese Materia Medica, Shanxi University of Chinese Medicine, Taiyuan 030619, China; 13145263888@163.com; 3 Engineering Research Center for Development and High Value Utilization of Genuine Medicinal Materials in North Anhui Province, School of Biological and Food Engineering, Suzhou University, Suzhou 234000, China

**Keywords:** Ziziphi Spinosae Semen flavonoid, anxiety, gut microbiota, amino acid metabolism, lipid metabolism

## Abstract

Ziziphi Spinosae Semen (ZSS), a homology of medicine and a type of seed, has been widely used to improve sleep quality. The present study aimed to assess the effects of ZSS flavonoid (ZSSF) extracted and isolated from ZSS on gut microbiota and hypothalamus metabolomic profiles in a chronic restraint stress (CRS)-induced anxiety mouse model. ZSSF was prepared using microporous resin chromatography, and seven compounds were determined by UPLC-MS. ZSSF treatment dramatically reduced anxiety-like behaviors, exerted sedative–hypnotic effects, increased hippocampal 5-HT and 5-HTP, and enhanced intestinal barrier function through inhibiting colon ZO-1, Claudin-1, and Occludin expression and reducing TNF-α, IL-6, and IL-1β levels. Compared with the CRS group, the diversity of gut microbiota in ZSSF-group mice was increased, with an increase in *Bacteroidetes* and a decrease in *Firmicutes*, and it was accompanied by an increase in fecal SCFAs. Hypothalamus metabolomics and lipidomics were performed to achieve 25 differential metabolites and 44 lipids, respectively. Serum metabolomics showed a total of 13 metabolites associated with anxiety were remarkably regulated by ZSSF. Weighted correlation network analysis (WGCNA) showed that glycerophospholipids (GPs) as well as phenylalanine, tyrosine, and L-tryptophan in peripheral and central parts were significant metabolites, which contributed to the pharmacological action of ZSSF. The mRNA expression of TPH2 and DDC key enzymes associated with tryptophan metabolism were upregulated, and PLA2G12A, LACT, and PLA2G6 key enzymes associated with GP metabolism were downregulated in ZSSF compared with CRS. Briefly, ZSSF improved tryptophan and GP metabolism and regulated the gut microbiome. This study may lay a theoretical basis for potentially developing ZSSF as a natural functional food ingredient for the improvement of anxiety and sleep disorders.

## 1. Introduction

The prevalence of anxiety disorders poses a significant global health challenge, affecting the lives of nearly 300 million individuals with various forms of anxiety disorders and exerting an impact on society as a whole [1]. Individuals with anxiety were also led to the subsequent development of other psychiatric comorbidities, such as depression, poor sleep quality, and even insomnia. To date, existing drugs for anxiety have had a minimal positive impact and are accompanied by drug-dependent side effects [2]. The efficacy of complementary and alternative therapies, including natural functional products, has been supported by a plethora of studies as a potentially safer and more efficacious approach for treating anxiety [3]. The increased consumption of natural functional products was a proposed approach for enhancing and maintaining overall health. One study has indicated that functional products have the potential to enhance the body’s capacity to resist chronic illnesses to an extent that is comparable to the efficacy of antioxidant supplements [4]. Therefore, alternatives to natural products associated with anxiety disorders are of great significance.

*Ziziphus jujube* Mill. var. *spinosa* (Bunge) Hu ex H.F. Chou. (ZJS), a plant belonging to the Rhamnaceae family, was widely distributed in the provinces of Shanxi, Hebei, Shaanxi, and Shandong in China and known as the “eastern sleep fruit”. Ziziphi Spinosae Semen (ZSS), the dried and ripe seed of ZJS, is an edible seed and has been most commonly used to improve poor sleep quality, anxiety-like mood disorders, and cardiovascular disease [5]. Our previous studies have demonstrated that the aqueous extract of ZSS could exert its sedative–hypnotic effect by the regulation of phenylalanine, tyrosine, and tryptophan biosynthesis pathways and neurotransmitter levels in p-chlorophenylalanine (PCPA)-induced insomnia rats [6,7]. Additionally, it has been reported that ZSS possesses the ability to counteract the disruption of gut microbiota caused by insomnia and enhance the levels of short-chain fatty acids (SCFAs) in fecal matter [8]. ZSS flavonoid (ZSSF) is the main active component of ZSS, accounting for 0.95%, mainly including spinosin and 6″′-feruloylspinosin. According to previous studies, spinosin exerted anxiolytic-like effects, and its action mechanism appeared to be regulated by GABA_A_ and 5-HT1_A_ receptors [9]. Spinosin also increased rapid eye movement (REM) sleep time and potentiated pentobarbital-induced sleep via a serotonergic mechanism [10]. It has been reported that the bioavailability of spinosin is only 2.2% [11,12]. However, whether ZSSF protected against chronic restraint stress (CRS)-induced anxiety and its potential mechanisms remain unclear. 

A previous study has proved that the microbiota–gut–brain axis (MGB axis) played a key role in neuropsychiatric disorders such as anxiety and depression [13]. The bidirectional relationship between the host brain and the microbiota involves interactions across various physiological pathways, including endocrine, neural, immune, and metabolic systems [14]. A clinical study confirmed that patients with generalized anxiety disorder (GAD) exhibited gut microbiota dysbiosis when compared to healthy controls [15]. Increased contents of harmful microbiota such as Bacteroidetes, Proteobacteria, and Actinobacteria in GAD patients and animals were observed [16]. What is more, SCFAs, the metabolites of the gut microbiota, may be seen as signaling molecules in the MGB axis [17]. For example, mice with anxiety-like behavior had lower levels of fecal acetic acid, propanoic acid, valeric acid, and isobutyric acid [18]. Meanwhile, the gut microbiota exerted a significant influence on both peripheral and central lipid metabolism in the host [19]. The dysregulation of the gut microbiota may impact amino acid and lipid metabolism, thereby contributing to the development of anxiety disorders [20]. Collectively, the regulation of the gut microbiota presented a promising strategy for mitigating anxiety disorders.

Therefore, in this study, we assessed the anti-anxiety activities of ZSSF on CRS-induced anxiety-like behavior in mice and explored the underlying mechanisms. Firstly, a CRS mouse model was used to mimic the pathogenesis and pathophysiology of anxiety-like behavior, and the anti-anxiety effects of ZSSF were evaluated. Secondly, weighted correlation network analysis (WGCNA) was applied on integrated gut microbiota, fecal SCFA levels, serum and hypothalamus metabolomics, as well as hypothalamus lipidomics to investigate the mechanism by which ZSSF exerted its anti-anxiety effects via the gut–brain axis. Finally, a verification experiment was conducted. This study may lay the foundation for ZSSF as a natural functional food for the improvement of anxiety disorder accompanied by insomnia.

## 2. Materials and Methods

### 2.1. Chemicals, Reagents, and Materials

The information on reference standards (flavonoid compounds, SCFAs, and lipid internal standards) is listed in Table S1. Formic acid and acetonitrile (LC-MS grade) were supplied by Fisher Scientific (Thermo Fisher Scientific, Waltham, MA, USA). All other reagents were of analytical grade.

ZSS (batch NO. 201909) was purchased from Anguo Jiarun Herbal Medicine Co., Ltd. (Anguo, China) and authenticated to be the dried seeds of ZJS by Prof. Chenhui Du from the Shanxi University of Chinese Medicine. The voucher specimens were preserved at the Modern Research Center for TCM, Shanxi University, Taiyuan, China. Diazepam (DZP) tablets, the positive control drug, were purchased from Shandong Xinyi Pharmaceutical Co., Ltd. (De zhou, China). Pentobarbital sodium salt was supplied by Merck (Merck KGaA, Darmstadt, Germany).

### 2.2. Extraction and Purification of ZSSF

Pulverized ZSS (2300 g) was immersed in petroleum ether (w:v, 1:10) overnight and then extracted two times under reflux (90 °C) for 1 h each time. After filtration through a filter paper, the residue was dried at 50 °C, soaked with 70% ethanol (*w*/*v*, 1:10) for 4 h, and extracted twice by heating reflux (2 h each time). The filtrate was combined and concentrated using a rotary evaporator to obtain a concentrated liquid with a concentration of 1 g/mL (according to the amount of crude drug). Furthermore, the concentrated liquid was extracted with water-saturated n-butanol 4 times (v:v, 1:1). After combining the extracts, the n-butanol parts were concentrated under reduced pressure (yield: 2.37%). Subsequently, the n-butanol fractions (0.5 g crude drug/mL) were subjected to chromatography on a D101 microporous resin column and eluted with 10 L of distilled water, followed by 10 L of 30% ethanol. The 30% eluted fractions were collected, concentrated, and freeze-dried to powder (the yield of 0.4%), named ZSSF. The contents of swertisin, vicenin II, kaempferol-3-*O*-rutinoside, spinosin, isovitexin, 6″′-feruloylspinosin, and rutin were simultaneously detected by UPLC-Q-Trap-MS/MS, and the detailed information and parameters are listed in Supplement File S1 and Table S2.

### 2.3. Animal Experimental Design and Sample Collection

Male ICR mice (Body weight: 18–20 g, 6–8 weeks of age) were supplied by Vital River Laboratory Animal Technology Co., Ltd. (Beijing, China, License No. SCXK (Jing) 2021-0006) and reared under standard conditions with a strict 12 h:12 h light/dark cycle, 50 ± 10% relative humidity, and 24 ± 2 °C temperature. Animal welfare and experimental operations were conducted in strict accordance with the Animal Ethics Committee of Shanxi University (registration number: SXULL 2021029).

Following acclimating for one week, mice were randomly allocated into the control group (CN) and the CRS-induced anxiety group (n = 15). In the anxiety group, mice were exposed to 3–4 h of confinement in 50 mL conical tubes with air holes per day for 14 consecutive days. The CRS mice were further randomly divided into four groups: (1) model (MD) group, (2) DZP group (positive control, 2 mg/kg), (3) low-dose ZSSF group (ZSSFL, 50 mg/kg), and (4) high-dose ZSSF group (ZSSFH, 100 mg/kg). Mice in the DZP, ZSSFL, and ZSSFH groups were intragastrically administered for three weeks. The CN and MD groups were administered an equal volume of 0.5% CMC-Na. The CRS procedure was conducted each day after drug administration for two weeks. At the end of CRS modeling, the mice were subjected to elevated plus maze (EPM) on the 15th and 16th days, a sleep test (ST) on the 17th and 18th days, and an open field test (OFT) on the 19th and 20th days, 1 hour after their dose. The specific methods of EPM and OFT are shown in Supplementary File S2. The experiment concluded with the collection of fresh fecal samples, which were subsequently stored at a temperature of −80 °C. Then, all mice fasted for 12 h and had their blood taken through removing the eyeballs, and they were sacrificed by cervical dislocation. The serum was separated by centrifugation at 3500 rpm for 15 min. The hippocampus and hypothalamus were promptly dissected from the brain and rapidly frozen in liquid nitrogen. All samples were stored at −80 °C until analysis.

### 2.4. Biochemical Assays

The corticosterone (CORT) and corticotropin-releasing hormone (CRH) levels in the serum, serotonin (5-HT) and 5-hydroxytryptophan (5-HTP) in the hippocampus, as well as tumor necrosis factor-α (TNF-α), interleukin (IL)-6, and IL-1β in the colon were quantified using ELISA kits (Table S1).

### 2.5. Histopathological and Immunofluorescence Analysis

The details of hematoxylin-eosin (H&E) of the hippocampus and colon, alcian blue-periodic acid-Schiff (AB-PAS), and immunofluorescence of the colon are shown in Supplementary File S3.

### 2.6. Measurement of SCFA Concentrations

The fecal SCFAs (acetic acid, propionic acid, butyric acid, valeric acid, and isobutyric acid) were determined on an Agilent 7890 B GC-MS coupled with an Agilent 5977A mass selective detector (Agilent, Santa Clara, CA, USA). Firstly, 40 mg of freeze-dried fecal samples were mixed with 250 μL water and homogenized at 60 Hz for 3 min. Then, the fecal homogenate was added to 20 μL of 0.1 mol/L sulfuric acid (pH ≈ 2), and the mixture sample was vortexed using 75% methanol (v:v) for 1 min. Afterward, the homogenized mixture was centrifuged at 13,000 rpm (4 °C, 10 min). Finally, 1100 μL of the supernatant was spiked with 6 μL of 2-ethybutyric acid (internal standard, IS) and 100 μL of methanol and then vortexed. The GC-MS conditions, reference solution preparation, and linearity are shown in Supplementary File S4. The quantification was performed by analyzing the data acquired in the timed selected ion monitoring (SIM) mode using the retention times (RT) and *m*/*z* values given in Table S3. The results of SCFAs were expressed as analyte μg/mg dried feces.

### 2.7. Gut Microbiota Analysis

The 16S rRNA analysis was conducted by Suzhou PANOMIX Biomedical Tech Co., LTD. (Suzhou, China). Microbial DNA was extracted from the colonic content of mice for sequencing analysis, targeting the V3-V4 hypervariable region of the 16S rRNA gene. The primers 338F (5′-ACTCCTACGGGAGGCAGCA-3′) and reverse primer 806R (5′-GGACTACHVGGGTWTCTAAT-3′) were utilized for amplification. Quantification of PCR products was performed using the PicoGreen dsDNA Assay Kit (Thermo Fisher Scientific, Waltham, MA, USA), followed by high-throughput pyrosequencing on an Illumina MiSeq platform (Illumina, Inc., San Diego, CA, USA).

### 2.8. Metabolomics Analysis

#### 2.8.1. Hypothalamus Untargeted Metabolomics and Lipidomics

According to Xuan’s method [21], biphasic extraction was used to obtain the polar part for untargeted metabolomics and the lipid part for lipidomics. Hypothalamus tissues (10 mg) were extracted using 300 μL pre-cold methanol including seven ISs (containing phosphatidylcholine (PC) 19:0/19:0 at 1.6 μg/mL, lysophosphatidylcholine (LPC) 19:0 at 0.8 μg/mL, phosphatidyl ethanolamine (PE) 17:0/17:0 at 0.8 μg/mL, ceramide (Cer) d18:1/17:0 at 0.5 μg/mL, triacylglycerol (TG) 15:0/15:0/15:0 at 1.25 μg/mL, and free fatty acid (FFA) 16:0_d3 at 0.8 μg/mL) by homogenization at 20 Hz for 2 min, followed by vortexing for 10 s, and then 1 mL MTBE was added. Subsequently, the mixture was vortexed for 15 min, and 300 μL of H_2_O was added and vortexed for 10 s to form a two-phase system. After standing for 15 min at 4 °C, the mixture was centrifuged at 13,000 rpm for 15 min at 4 °C. The upper phase (800 μL) was concentrated in nitrogen in a Termovap Sample Concentrator (Biotage, Uppsala, Sweden), and the residues were redissolved using 50 μL ACN/IPA/H_2_O (65:30:5, *v*/*v*/*v*) containing 10 mM ammonium acetate to obtain the lipid part for lipidomics. In addition, 500 μL of the bottom phase (the polar part) was transferred, concentrated, and redissolved using 60 μL water/methanol (8:2, *v*/*v*) for the untargeted metabolomics. The supernatant above was obtained by centrifuging at 13,000 rpm for 15 min at 4 °C for LC-MS analysis. Details of chromatographic separations, mass parameters, and data analysis are provided in Supplementary Files S5 and S6. Detailed information on lipid internal standards (ISs) and lipid classification are listed in Table S4.

#### 2.8.2. Serum Untargeted Metabolomics

The serum sample pretreatment was similar to our previous method [22]. Briefly, the serum sample (50 μL) was mixed with pre-cooled methanol-acetonitrile (1:1, 200 μL) for protein precipitation, vortexed for 1 min, and then centrifugated (13,000 rpm, 15 min, 4 °C). The supernatants were transferred and dried using a SpeedVac system (Eppendorf, Hamburg, Germany), and then each sample was dissolved in 100 μL 50% methanol and vortexed for 3 min. The supernatant was transferred to an autosampler vial for UPLC-Q-TOF/MS analysis. Details of chromatographic separations and mass parameters are provided in Supplementary File S7. The data processing method was the same as that of hypothalamus metabolomics.

#### 2.8.3. Preparation of Quality Control (QC) Sample

The stability of analytical methods and instruments was investigated by preparing QC samples through the combination of 5 μL from each analyzed sample. Furthermore, QCs were injected six times prior to the batch process and then injected once every six samples throughout the entire analysis procedure.

### 2.9. Reverse Transcription-Quantitative PCR (RT-qPCR) Experiment

RNA was extracted from mouse brain tissue using a MonPure universal RNA kit (Yeasen Biotechnology Co., Ltd., Shanghai, China) Then, RNA was reverse transcribed to cDNA using a reverse transcription kit (TaKaRa, Kusatsu, Japan). SYBR Green real-time PCR amplification and detection were performed using a TIANLONG Real-Time PCR System (Xi’an Tianlong Science and Technology Co., Ltd., Xi’an, China). The relative expression of mRNA was calculated using the 2^−ΔΔCt^ method with GAPDH as the reference gene. The forward and reverse primers of TPH2, DDC, PLA2G6, LCAT, PLA2G12A, TNF-α, IL-1β, IL-6, and GAPDH are displayed in Table S5.

### 2.10. Statistical Analysis

Multivariate statistics were conducted using SIMCA software package v14.0 (Umetrics; Umea, Sweden). One-way ANOVA was conducted to determine differences among groups, and Student’s *t*-test was used to analyze the differences between the two groups. Data analysis was performed using SPSS version 25.0 (International Business Machines Corporation, Armonk, NY, USA) and GraphPad Prism version 9.0 (GraphPad Software, San Diego, CA, USA) software. The weighted correlation network analysis (WGCNA) was conducted to identify key serum and hypothalamus metabolic modules related to anxious phenotypes. Pearson correlation analysis values of |r| > 0.7 and *p* < 0.05 were considered significant.

## 3. Results

### 3.1. Quantitative and Qualitative Analysis of Seven Compounds in ZSSF by UPLC-Q-Trap-MS/MS

**Figure 1:**
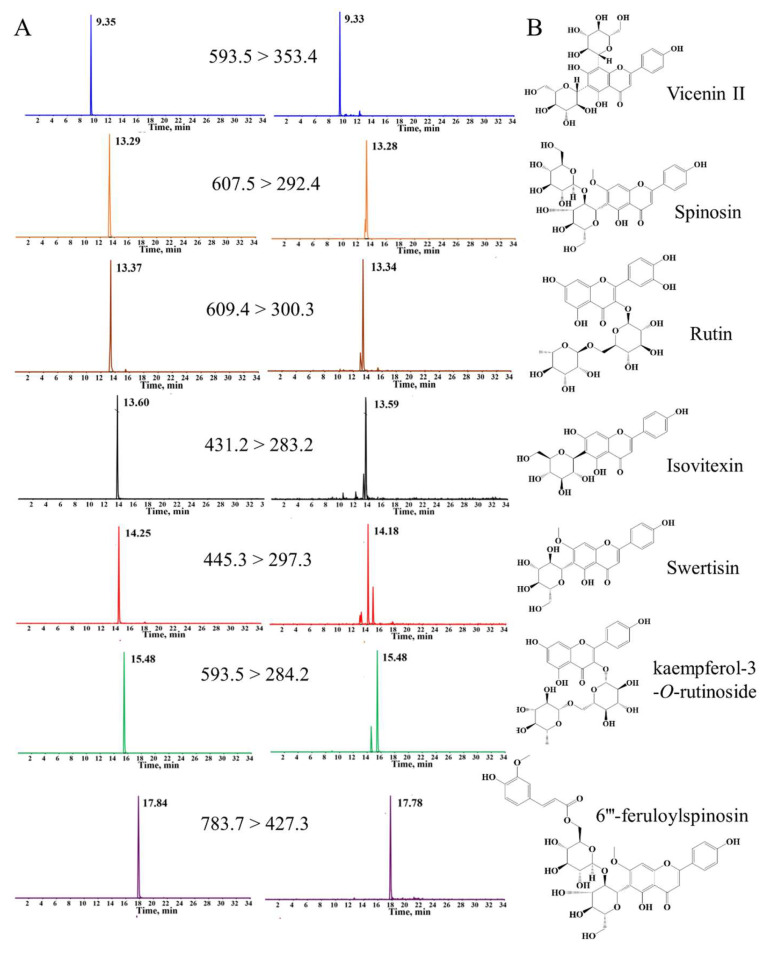
(**A**) The MRM chromatograms. (The left chromatograms represent seven references; the right chromatograms represent seven compounds in samples). (**B**) Chemical structure of seven compounds.

In the present study, the multiple reaction monitoring (MRM) information-dependent acquisition (IDA)-enhanced product ion (EPI) scanning mode was applied for simultaneous quantitative and qualitative analyses of seven compounds in ZSSF. Firstly, retention times and MS2 spectra generated by the MRM-IDA-EPI mode were matched to confirm the seven compounds by comparing them with reference standards. The MRM spectra and the chemical structures of seven compounds in ZSSF are shown in Figure 1A and in Figure 1B, respectively. As shown in Table S6, the linear regression of all compounds within the test ranges exhibited excellent performance (r > 0.9995). The LOQs (0.025–1.5 μg/mL) were appropriate for quantitative detection. The relative standard deviations (RSDs) of all analytes of the intra-day and inter-day variations were less than 2.80% and 2.96%, respectively. In the repeatability test, the RSDs of seven compounds were less than 2.94%. Meanwhile, the RSD values for stability were less than 2.85% for all analytes, indicating that the sample solutions were stable within 24 h, and the analytical method was accurate, with recoveries of 92.03–106.11%. The validated method was utilized for the simultaneous determination of seven compounds. The average content of swertisin, vicenin II, kaempferol-3-O-rutinoside, spinosin, isovitexin, 6″′-feruloylspinosin, and rutin in ZSSF from three batch samples were calculated as 0.20 ± 0.005% (*w*/*w*), 0.97 ± 0.018% (*w*/*w*), 1.3 ± 0.015% (*w*/*w*), 21.4 ± 0.17% (*w*/*w*), 0.19 ± 0.05% (*w*/*w*), 7.8 ± 0.12% (*w*/*w*), and 0.4 ± 0.01% (*w*/*w*), respectively.

### 3.2. ZSSF Improved Anxiety-like Behavior and Exerted Sedative–Hypnotic Effects in CRS-Induced Mice

**Figure 2:**
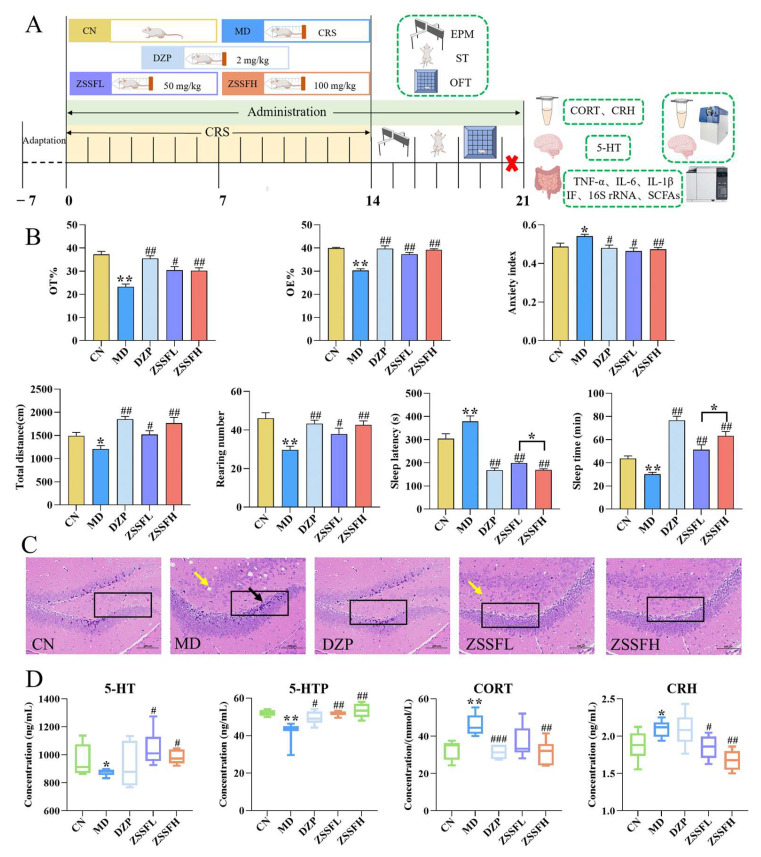
Anti-anxiety effects of ZSSF on CRS-induced anxiety-like behavior in mice. (**A**) Animal experimental protocol. (**B**) Behavioral index of mice in EPM, OFT, and ST (n = 9–12). (**C**) H&E staining. (**D**) The level of hippocampal 5-HT, 5-HTP, and serum CORT and CRH. Data expressed as mean ± SEM. * *p* < 0.05, ** *p* < 0.01 versus the CN group; # *p* < 0.05, ## *p* < 0.01, ### *p* < 0.001 versus the MD group. The cross sign indicated the euthanasia of mice. The black box represented the DG area. Black arrows indicated darker staining of cells and the yellow arrowed indicate that the cells are loosely arranged.

The behavior experimental design is shown in Figure 2A. As shown in Figure 2B, the OT%, OE%, total distance traveled, and rearing number of the mice exposed to the CRS paradigm exhibited significant reductions (*p* < 0.05 or *p* < 0.01), and a significant increase (*p* < 0.05) was found in the anxiety index, suggesting the successful induction of anxiety-like phenotypes in CRS mice. In contrast, DZP treatment regulated the values of these parameters compared with the MD group. Similarly, ZSSFL and ZSSFH significantly reversed these five indexes in CRS mice (*p* < 0.05). Importantly, ZSSFH showed similar effects to DZP. Consistent with our previous results, CRS mice exhibited shorter sleep times (*p* < 0.01) and longer sleep latency (*p* < 0.05) when compared with the CN group [22]. Meanwhile, ZSSFH exhibited stronger sedative–hypnotic effects than the ZSSFL group (*p* < 0.05). The above behavioral results suggest that ZSSF treatment dramatically reduced anxiety-like behaviors in CRS-treated mice and exerted sedative–hypnotic effects.

Compared to the CN group, neurons in the hippocampal DG regions in the MD group were arranged loosely and disordered, accompanied by deeper cell stains (Figure 2C). Three drug treatment groups could dramatically alleviate the above-mentioned histopathological damages. As shown in Figure 2D, the CRS mice showed significant reductions in hippocampal 5-HT and 5-HTP (*p* < 0.05 or *p* < 0.01), which were significantly reversed by ZSSFL and ZSSFH (*p* < 0.05 or *p* < 0.01). DZP exhibited no obvious changes in hippocampal 5-HT, in the MD group. The serum CORT and CRH levels in CRS-induced mice were significantly elevated compared with the CN group (*p* < 0.01 or *p* < 0.05). ZSSFH significantly decreased the levels of serum CORT and CRH in the CRS-treated mice, while ZSSFL had no decreasing effect on the CORT, only reversing the level of CORT. Consistent with the above pharmacodynamic results, ZSSFH had better regulatory effects on the suppression of the HPA axis and hippocampal 5-HT induced by CRS than ZSSFL, so ZSSFH was selected for a subsequent mechanism study.

### 3.3. ZSSFH Restored the Intestinal Mucosal Barrier Function

**Figure 3:**
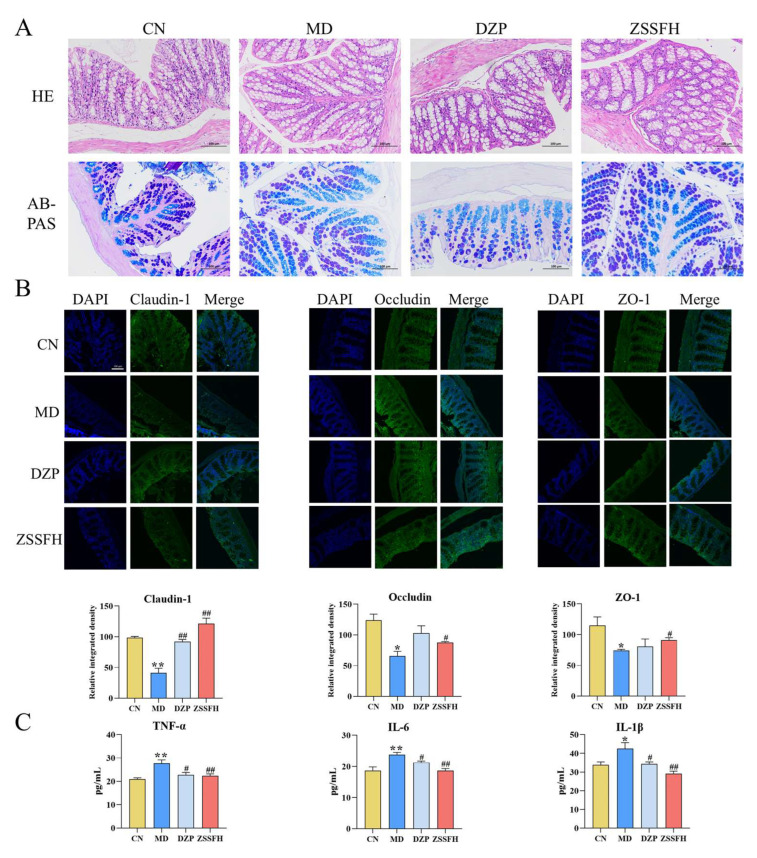
Protective effects of ZSSFH against intestinal mucosal barrier damage in CRS-induced anxiety in mice. (**A**) HE and AB-PAS staining. (**B**) Images of immunofluorescence staining and integrated density analysis of Claudin, Occluding, and ZO-1 in the colon tissues. (**C**) The level of colonic TNF-α, IL-6, and IL-1β. Data expressed as mean ± SEM. * *p* < 0.05, ** *p* < 0.01 versus the CN group; # *p* < 0.05, ## *p* < 0.01 versus the MD group.

As shown in Figure 3A, four groups had no significant differences in colonic morphological damage and the number of goblet cell counts by HE staining and AB-PAS. The effects of ZSSFH and DZP on intestinal mucosal barrier disruption and inflammatory factor levels in the colon were also analyzed. The integrated density analysis of immunofluorescence staining showed the potential of ZSSFH to reverse the decreased levels of Claudin-1, Occludin, and ZO-1 in the colon tissues of CRS mice (Figure 3B). However, DZP only reversed Claudin-1. As shown in Figure 3C, the levels of TNF-α, IL-6, and IL-lβ were significantly elevated in the colons of the MD group compared to the CN group. ZSSFH and DZP treatments decreased TNF-α, IL-6, and IL-1β to levels comparable to those in the CN group. Although no obvious histological damage was induced, CRS resulted in intestinal barrier disruption and inflammation in mice, which could be reversed by ZSSFH.

### 3.4. ZSSFH Alleviated CRS-Induced Gut Dysbiosis

#### 3.4.1. ZSSFH Improved the Diversity of the Gut Microbiome

**Figure 4:**
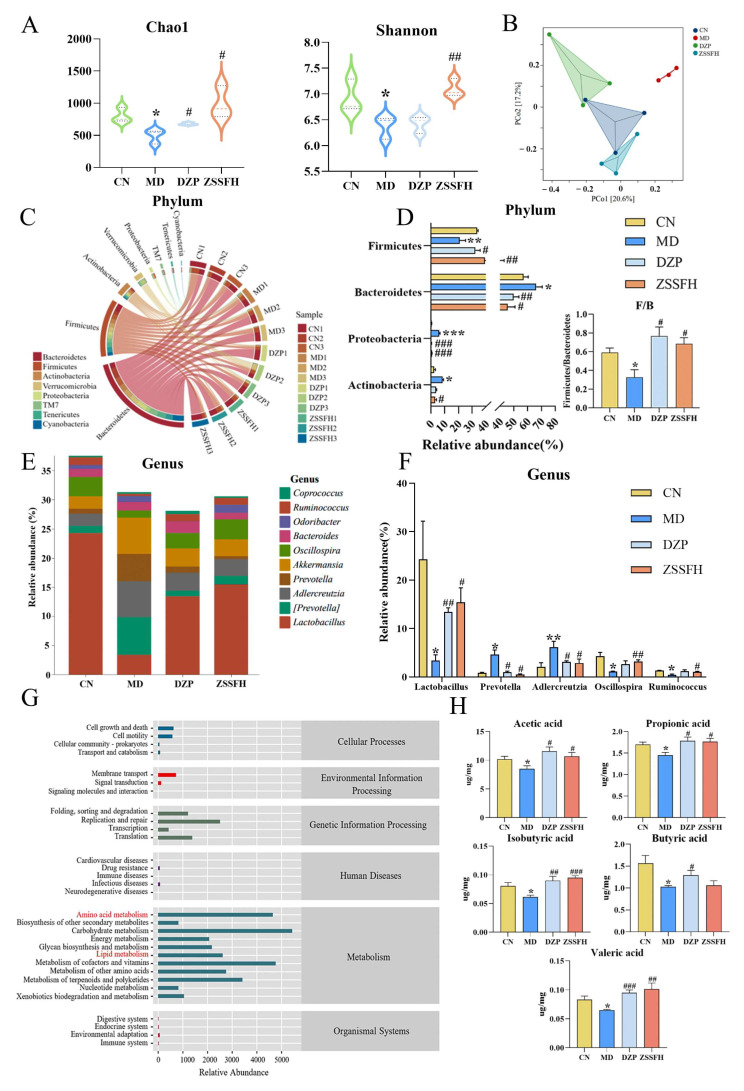
Regulation of ZSSFH on the gut microbiota structure in CRS-induced anxiety-like behavior in mice. (**A**) α-diversity index based on Chao1 and Shannon. (**B**) Principal coordinate analysis (PCoA) with Bray–Curtis distance. (**C**) The Circos plot showing the correlation between representative phylum and different groups. (**D**) Relative abundance of Firmicutes, Bacteroidetes, Proteobacteria, and Actinobacteria and the ratio of Firmicutes/Bacteroidetes (F/B) at the phylum level. (**E**) Bar plot of bacterial richness distribution at the genus level. (**F**) Relative abundance of Lactobacillus, Prevotella, Adlercreutzia, Oscillospira, and Ruminococcus. (**G**) PICRUSt2 prediction analysis. (**H**) Effect of ZSSFH on fecal SCFAs. Significant correlations are marked by * *p* < 0.05, ** *p* < 0.01, *** *p* < 0.001 versus the CN group; # *p* < 0.05, ## *p* < 0.01, ### *p* < 0.001 versus the MD group.

We conducted the 16S rRNA sequencing to assess the impact of ZSSFH on gut microbiota structures in colonic content. As compared with the CN group, the MD group’s richness (Chao 1) and diversity (Shannon) indexes were significantly decreased (*p* < 0.05 and *p* < 0.01, respectively). ZSSFH treatment significantly increased the two indexes (Figure 4A). Moreover, a principal coordinate analysis (PCoA) revealed distinct clustering of the microbiota composition for CN, MD, and two drug treatment groups (Figure 4B).

#### 3.4.2. ZSSFH Changed the Composition of the Gut Microbiota and Regulated Amino Acid and Lipid Metabolism

At the phylum level, the dominant bacteria among four groups were Bacteroidetes, Firmicutes, Actinobacteria, Verrucomicrobia, and Proteobacteria (>90%, Figure 4C). The relative abundances of Bacteroidetes, Proteobacteria, and Actinobacteria were notably increased, while the relative abundances of Firmicutes and the ratio of Firmicutes/Bacteroidetes (F/B) were significantly decreased in the MD group in comparison with the CN group (Figure 4D, >30%). Meanwhile, ZSSFH significantly reversed Firmicutes, Bacteroidetes, Actinobacteria, and Proteobacteria, and DZP only regulated three of them. At the genus level, the bacteria predominantly consisted of Lactobacillus (Firmicutes phylum, >20%), Prevotella (Bacteroidetes phylum, >5%), Adlercreutzia (Actinobacteria phylum, >5%), Oscillospira (Firmicutes phylum, >5%), and Ruminococcus (Firmicutes phylum, >3%) (Figure 4E). The decreased relative abundances of Lactobacillus, Oscillospira, and Ruminococcus and increased relative abundance of Prevotella and Adlercreutzia in the MD group were observed (Figure 4F). Comparatively, ZSSFH notably reversed the above changes, and DZP only regulated Lactobacillus, Prevotella, and Adlercreutzia changes. Likewise, we observed variations at the family level, and the 10 most abundant bacteria (> 80%) were enriched and shared by all groups (Figure S1A). As shown in Figure S1B, the higher relative abundances of S24-7 (Bacteroidetes phylum), as well as lower relative abundances of Lactobacillaceae (Firmicutes phylum) and Ruminococcaceae (Firmicutes phylum), in the MD group were observed. Notably, ZSSFH significantly decreased the relative abundance of S24-7 and increased Lactobacillaceae and Ruminococcaceae (*p* < 0.05, *p* < 0.01). DZP significantly reversed the relative abundances of S24-7 and Lactobacillaceae. Together, Firmicutes and Bacteroidetes were major microbiota related to ZSSFH treatment.

Finally, PICRUSt2 function prediction based on the KEGG enrichment analysis was conducted. The result revealed that the effects of ZSSFH on anxiety-related metabolism on the top five clustered on amino acid, lipid, and carbohydrate metabolism as well as the metabolism of cofactors and vitamins and the metabolism of terpenonids and polyketides (Figure 4G).

#### 3.4.3. ZSSFH Increased the Concentration of Fecal SCFAs

Fecal SCFA content was determined by GC-MS. A linear regression equation was established (Table S7) and successfully applied to determine five SCFAs from all groups (Figure S2). As shown in Figure 4H, a comparison between the MD and CN groups showed a significant reduction in the levels of acetic acid, propionic acid, butyric acid, valeric acid, and isobutyric acid in the MD group (*p* < 0.05). ZSSFH treatment statistically increased the concentration of SCFAs except for butyric acid.

### 3.5. ZSSFH Regulated the Hypothalamus Amino Acid and Glycerophospholipid (GP) Metabolism in CRS Mice

**Figure 5:**
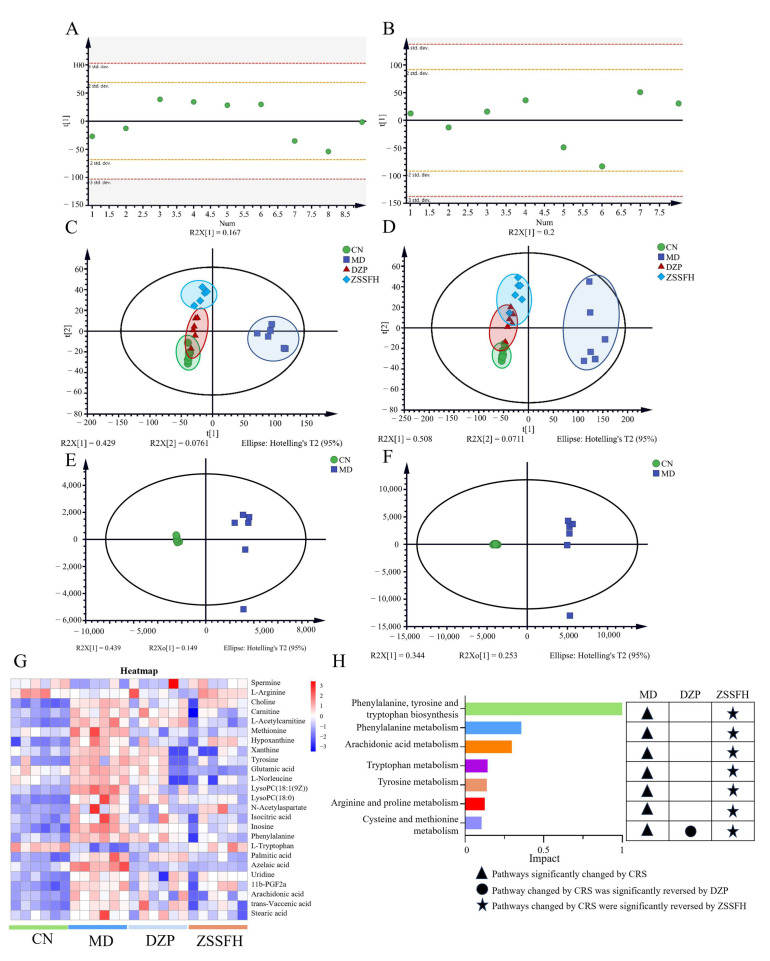
ZSSFH treatment regulated hypothalamus metabolic profiles in CRS-induced mice. RSD distribution of QC samples for evaluating the method in the positive ion mode (**A**) and negative ion mode (**B**). PLS-DA score plot of four groups in the positive (**C**) and negative ion mode (**D**). Score plot of OPLS-DA model based on par scaling for MD and CN separation in the positive ion mode (**E**) and negative ion mode (**F**). Heat map of 25 metabolites (**G**). Metaboanalyst pathway analysis of differential metabolites of mice (**H**).

By using UPLC-Q-TOF/MS, untargeted metabolomics was conducted on the hypothalamus and serum samples from four groups. For hypothalamus metabolomics, all QC samples in the polar part were within twice the SD in the score map, indicating the good stability of the instrument (Figure 5A,B). The partial least squares discrimination analysis (PLS-DA) score plot showed clear separation among CN, MD, and two drug treatment groups. In both positive (Figure 5C) and negative (Figure 5D) ion modes, DZP and ZSSFH showed a closer tendency to the CN group, whereas MD was far from them, suggesting that DZP and ZSSFH could modify hypothalamus metabolic disturbances in CRS mice. Subsequently, a supervised orthogonal partial least squares discrimination analysis (OPLS-DA) was used to find the different metabolites between the CN and MD groups (Figure 5E,F), and the model quality parameters are listed in Table S8. A total of 25 metabolites were identified, of which 22 metabolites were increased, and 3 metabolites were decreased in the MD vs. CN group; 3 metabolites were upregulated, and 16 metabolites were downregulated in the ZSSFH group vs. MD group; 10 metabolites were downregulated in the DZP group vs. MD group (Table S9, Figure 5G). As shown in Figure 5H, the disturbance of hypothalamic metabolites in mice with anxiety induced by CRS was related to amino acid metabolism, which was all regulated by ZSSFH. But, DZP only reversed cysteine and methionine metabolism.

**Figure 6:**
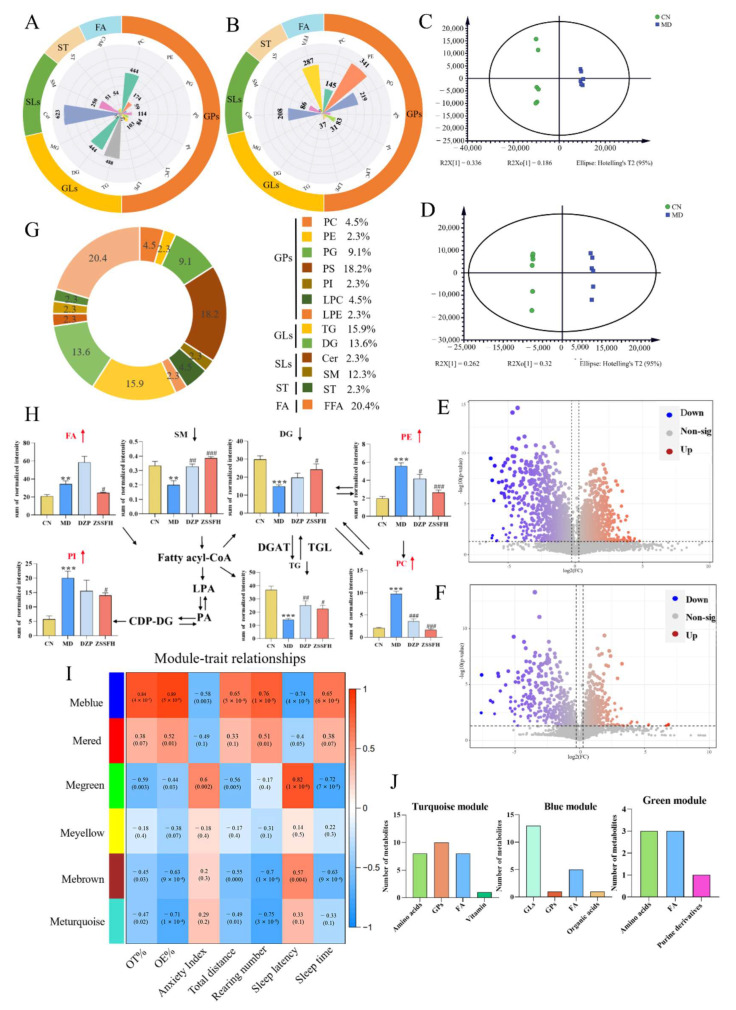
ZSSFH treatment regulated hypothalamus lipid metabolic profiles in CRS-induced mice. Numbers of lipids in peak lists under the positive ion mode (**A**) and negative ion mode (**B**). OPLS-DA score plot of CN and MD groups (**C**) and the negative ion mode (**D**); the volcano map of differential metabolites in the MD vs. CN group under the positive ion mode (**E**) and negative ion mode (**F**); the percentage of different metabolites in different lipid subclasses (**G**). Significantly changed lipids in related pathways (**H**). The WGCNA analysis of serum, hypothalamus differential metabolites and phenotype, and red and blue squares indicated positive and negative correlation, respectively (**I**). The number of metabolites in each module that is mainly significantly correlated with host phenotypes (**J**). Significant correlations are marked by ** *p* < 0.01, *** *p* < 0.001 versus the CN group; # *p* < 0.05, ## *p* < 0.01, ### *p* < 0.001 versus the MD group.

In the metabolic spectrum of lipidomics, a total of 4409 lipids were involved, including five classes of glycerophospholipids (GPs), glycerolipids (GLs), sphingolipids (SLs), sterols (ST), and fatty acid (FA). They covered 15 lipid subclasses: PC, phosphatidylinositol (PI), phosphatidylserine (PS), LPC, LPE, Cer, diglycerides (DG), TG, sphingomyelin (SM), sterols (ST), and carnitine (Car) under the positive ion mode and PE, Cer, phosphatidylglycerol (PG), and FFA under the negative ion mode (Figure 6A,B). A total of 90.4% and 96.2% (positive and negative ion modes) of lipid molecular species possessed an RSD of less than 30% in QC samples, showing that the whole analysis process had good repeatability and stability (Figure S3A,B). OPLS-DA was used to identify the metabolic profile differences between CN and MD groups (Figure 6C,D, Table S8). The volcano map (Figure 6E,F), combined with VIP, p, and FC, was applied to find different metabolites. Finally, a total of 44 lipids were identified and classified as five lipid classes according to neutral loss (NL) and lipid head base fragments (Figure 6G, Table S10). As shown in Figure 6H, the relative contents of PC, PE, PI, and FA were significantly increased, while SM, DG, and TG were decreased in the MD group in comparison with the CN group. All seven disturbed lipids were significantly reversed after ZSSFH treatment. However, DZP only regulated SM, PE, PC, and TG. These results show the advantageous effect of ZSSFH on the regulation of brain lipid and amino acid metabolism, which is greater than that of DZP.

In serum, the metabolic profile, and different metabolites were evaluated using the same method (Figure S3C–J). Treatment with ZSSFH regulated 13 metabolites, including methionine, tyrosine, L-tryptophan, LysoPC (18:1(9Z)), palmitoleic acid, dihomo-gamma-linolenic acid (DGLA), 10Z-nonadecenoic acid, citric acid, glycerol-3-phosphate, eicosapentaenoic acid (EPA), docosahexaenoic acid (DHA), arachidonic acid (AA), and oleoyl-L-α-lysophosphatidic acid (LPA). The altered metabolism pathways were phenylalanine, tyrosine, and tryptophan biosynthesis and arachidonic acid, tyrosine, and tryptophan metabolism (Figure S3K,L). However, DZP significantly regulated only three metabolism pathways.

Finally, we performed a WGCNA analysis of 91 differential amino acid and lipid metabolites identified from serum and hypothalamus samples. In total, six modules were identified based on the best topological overlap matrix with a soft-thresholding power of seven (Figure 6I). Among them, the blue module, green module, and turquoise module were notably associated with at least two kinds of phenotype (|r| > 0.7, *p* < 0.05). As shown in Figure 6J, the blue module, the largest one, was significantly correlated with six phenotypes. Peripheral and central GLs and FA were the main lipid metabolites in this module. In addition, GPs, FA, and amino acids were mainly involved in the turquoise module and the green module. Hence, GPs, GLs, FA, and amino acid metabolites in phenylalanine, tyrosine, and tryptophan biosynthesis and tryptophan and GP metabolism in peripheral and central parts were significant metabolites, which contributed to the pharmacological action of ZSSFH.

### 3.6. Correlation Analysis Between Altered Microbial Genera and Behavior Index, 5-HT, and 5-HTP

**Figure 7:**
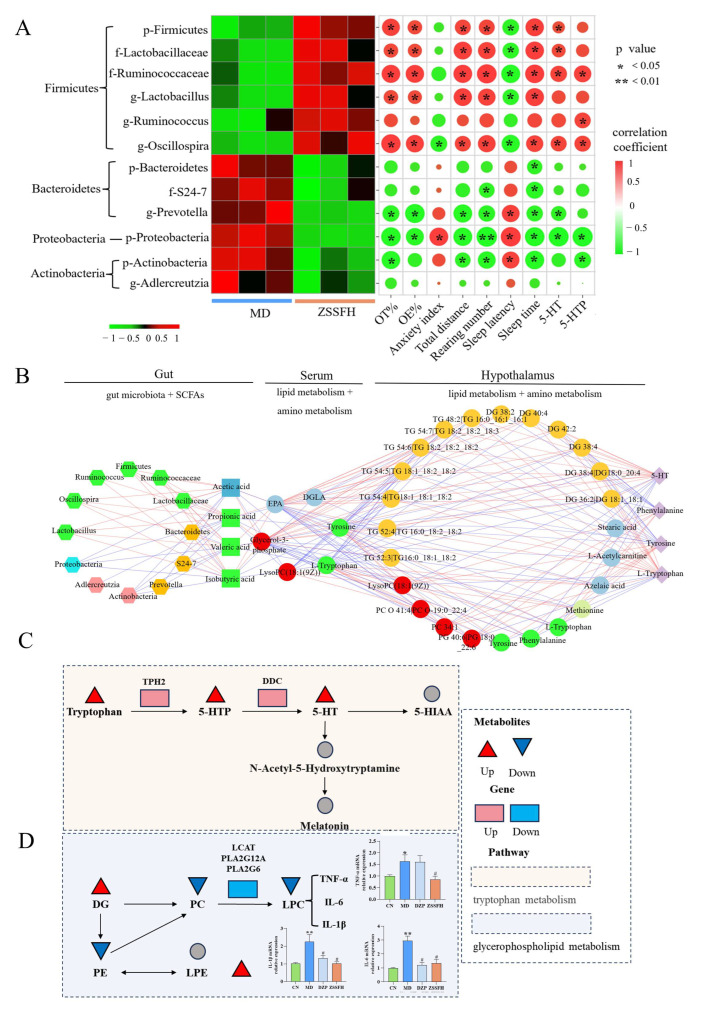
(**A**) Correlations between differential gut microbiota and anxiety-related phenotypes as well as 5-HT and 5-HTP. The r value is represented by the gradient color, with red indicating a positive correlation and green indicating a negative correlation. Significant correlations are marked by * *p* < 0.05, ** *p* < 0.01 versus the CN group; # *p* < 0.05 versus the MD group. (**B**) Possible ways connecting the gut and brain in CRS-induced anxiety-like behavior associated with ZSSF. All lines showed significant correlations, with red indicating a positive correlation and blue indicating a negative correlation. In gut bacteria, green indicated Firmicutes phylum, orange indicated Bacteroidetes phylum, pink indicated Actinobacteria phylum, and blue indicated Proteobacteria phylum. In serum and hypothalamus differential metabolites, red represented GPs, green represented amino acid, and blue represented FA. (**C**) The mRNA expression of TPH2 and DDC in the brain. (**D**) The mRNA expression of PLA2G12A, LCAT, PLA2G6, TNF-α, IL-6, and IL-1β in the brain.

Firstly, we used Pearson’s correlation analysis to explore the relationship between anxiety and gut microbiota amplicon sequence variants (ASVs). As shown in Figure 7A, p-Firmicutes, f-Lactobacillaceae, f-Ruminococcaceae, g-Lactobacillus, and g-Oscillospira were positively correlated with behavioral indexes (OT%, OE%, total distance and rearing number, and sleep time) and negatively correlated with sleep latency. In contrast, p-Proteobacteria and g-Prevotella were negatively correlated with OT%, OE%, rearing number, total distance, sleep time, and 5-HT and positively correlated with anxiety index and sleep latency. Additionally, p-Actinobacteria exhibited a significant negative correlation with OT%, total distance, rearing number, sleep time, and 5-HTP. Collectively, 5 of 12 (42%) taxa from the phylum, genera, and family were mainly assigned to the phylum Firmicutes, and 2 of 12 (16.7%) were assigned to the phylum Bacteroidetes, which were significantly correlated with anxiety.

Secondly, we found that 50% of the taxa (10/20) belonged to the Firmicutes phylum and positively correlated with propionic acid, valeric acid, and isobutyric acid (r = 0.978, 0.931, 0.929; *p* = 0.0007, 0.006, 0.007) (Table S12, Figure 7A). Furthermore, we explored the potential correlation between SCFAs and serum different metabolites. As shown in Figure 7B, propionic acid, valeric acid, and isobutyric acid were negatively correlated with GPs (LPC 18:1(9Z) and glycerol-3-phosphate) and amino acids (tryptophan and tyrosine) (r > 0.9, Table S13). In addition, those differential serum metabolites were significantly positively correlated with hypothalamic LysoPC (18:1(9Z)) and PC 34:1 as well as phenylalanine, tyrosine, tryptophan, 5-HT, and 5-HTP.

Finally, the genes related to tryptophan metabolism in brain tissue were determined (Figure 7C,D). In the MD group, the transcript levels of TPH2 and DDC genes were significantly decreased, and the transcript levels of PLA2G12A, LACT, and PLA2G6 related to GP metabolism were significantly increased, and the TNF-α, IL-6, and IL-1β genes were obviously enhanced, compared to the CN group (*p* < 0.05 or *p* < 0.01). Compared with the MD group, ZSSFH brain tissue had a significantly higher relative expression of TPH2 and DDC and lower transcript levels of PLA2G12A, LACT and PLA2G6, TNF-α, IL-6, and IL-1β (*p* < 0.05 or *p* < 0.01).

## 4. Discussion

ZSSF was the main active component, accounting for 0.95% of ZSS, with spinosin and 6″′-feruloylspinosin as the main components. The anxiolytic-like effects of spinosin have been reported previously, and its mechanism of action appeared to be modulated by 5-HT_1A_ receptors and GABA_A_ receptors [23]. Hence, we used an optimized method to purify ZSSF from ZSS using microporous resin chromatography, and the compounds in ZSSF were clearly identified and quantitatively characterized. In this study, we found that ZSSFH exhibited equal or even better anxiolytic and sedative–hypnotic effects than DZP. Over 3 weeks of treatment, ZSSFH could reduce serum CORT and CRH levels and suppress the hyperactivity of hippocampal 5-HT and 5-HTP. According to these results, ZSSF might be an effective therapeutic agent for anxiety accompanied by insomnia.

It has been reported that the bioavailability of spinosin was only 2.2% [11,12]. The results of our study suggested that ZSSF exerted anxiolytic effects through gut microbiota, which provided novel mechanistic insights into the mechanism of action of ZSSF with low oral bioavailability. The gut microbiota has been previously reported to have the ability to influence the host’s mood, while stress can independently shape the composition of the gut microbiota [24]. In the present study, CRS significantly altered the alpha and beta diversity, which corroborated the results of prior investigations [25,26]. In contrast, supplementation with ZSSFH maintained microbial diversity against CRS-induced gut dysbiosis. Gut microbiota, with *Bacteroidetes* and *Firmicutes* dominating, has been linked to the occurrence of depressive and anxiety disorders [13,15,27]. GF mice showed reduced anxiety-like behavior [28]. In addition, a shift toward an increase in the F/B ratio was identified in CRS mice. ZSSFH regulated dominant disturbed bacteria at the phylum level, including Bacteroidetes, Firmicutes, Proteobacteria, Verrucomicrobia, and Actinobacteria. Interestingly, Lactobacillus, Oscillospira, Ruminococcus, Prevotella, and Adlercreutzia were the predominant genera responding to ZSSFH treatment, which were also assigned to Firmicutes, Bacteroidetes, and Actinobacteria. Previous studies suggested that Bacteroides was more abundant than Firmicutes in GAD patients compared with healthy controls, which is consistent with our experimental results [13,15]. In the rats exhibiting anxiety-like behaviors, it was observed that the proportion of Firmicutes declined, while that of Bacteroides rose [26]. At the family level, ZSSFH treatment could decrease the abundance of Bacteroidetes S24-7 and increase the abundance of Lactobacillaceae (the Firmicutes phylum) and Ruminococcaceae (the Firmicutes phylum), which has been shown to be negatively related to anxiety-like behavior [29]. After 48 h of fermentation with fecal samples from healthy individuals, ZSSF was found to enhance *Firmicutes* enrichment in fecal bath culture. Pearson’s correlation analysis further showed that Firmicutes and Bacteroidetes played an important role in the anti-anxiety and anti-insomnia effects of ZSSFH. SCFAs, the primary metabolites derived from gut microbiota, assisted in maintaining the integrity of the gut barrier, with physiological concentrations having demonstrable effects on gut barrier function [30]. Firmicutes could effectively maintain the concentrations of propionic acid, valeric acid, and isobutyric acid, resulting in enhancing the expression of TJ proteins such as Claudin, Occludin, and ZO-1 in the colon, thereby decreasing gut permeability [31]. In the present study, increased *Firmicutes* abundance was significantly related to higher fecal acetic acid, propionic acid, valeric acid, and isobutyric acid. Also, we observed that ZSSF increased colon TJ protein expression and reversed colonic TNF-α, IL-6, and IL-1β levels. Together, ZSSF treatment could effectively maintain the abundance of Firmicutes and Bacteroidetes and the F/B ratio and modulate the gut microbiome toward a healthier profile.

Numerous studies have shown that the gut microbiota composition affects host metabolism by altering host metabolomes. The PICRUSt2 prediction of gut microbiota suggested that CRS mainly altered amino acid and lipid metabolism in the ZSSFH group. Clinical studies and animal experiments have shown that phenylalanine, tyrosine, and tryptophan biosynthesis and tryptophan metabolism were the disturbed metabolism pathways in anxiety [32,33,34]. Importantly, anxiety and depression behavior often coexist with lipid metabolism disorder [35]. Here, we found the metabolic pathways involving phenylalanine, tyrosine, and tryptophan biosynthesis, tryptophan metabolism, and GP metabolism were strongly correlated to anxiety and therapeutic interventions of ZSSF using WGCNA. Interestingly, the altered metabolic pathways showed an overlap for tryptophan and GP metabolism in the serum and hypothalamus for ZSSF but not for DZP.

The brain membrane lipids were composed of GPs (PC, LPC, PE, and so on), SM, and ST [36]. Disruptions in lipid metabolism had been observed in the prefrontal cortex of rats subjected to CRS [9]. Xu et al. found that more than 55% of lipid differential metabolites were detected in the hypothalamuses of mice with anxiety- and depressive-like behavior [37]. Similarly, we observed the increased hypothalamic levels of PC, PE, PI, and FA, as well as the decreased levels of SM, DG, and TG in CRS mice. Importantly, these changed lipids were reversed by ZSSFH. A previous study showed that PC and LPE were increased and SM was decreased in the brain of a chronically stress rat, and CORT serum levels were correlated with LPC and inversely correlated with SM [18,38]. In addition, the levels of serum LPC18:1(9Z), lysophosphatidic acid (LPA), dihomo-gamma-linolenic acid (DGLA), and arachidonic acid (AA) were increased, while the levels of eicosapentaenoic acid (EPA) and docosahexaenoic acid (DHA) were decreased in the MD group compared with the CN group, hinting that abnormal lipid metabolism occurred in CRS mice, which was in agreement with our previous study [22]. Here, we further demonstrated that serum tyrosine, tryptophan, and AA and LPC18:1(9Z), which were significantly enriched by the treatment of ZSSFH, had a positive association with higher hypothalamic tyrosine, tryptophan, AA and LPC18:1(9Z), 5-HT, and 5-HTP levels. In this study, the levels of 5-HT and 5-HTP and the mRNA expression of TPH2 and DDC in the hypothalamus were increased by ZZSFH. Tryptophan was first converted into 5-HTP by TPH2 in the brain and then decarboxylated by the DDC into 5-HT. Previous studies showed that the gut microbiota might affect the brain’s 5-HT levels by influencing the metabolism of tryptophan [39,40]. Xie et al. discovered significant relationships between tryptophan and bacterial taxa under *Firmicutes*, particularly the *Lactobacillus* genus [41]. The disorder of Lactobacillus led to a significant reduction in tryptophan metabolism. This, in turn, caused the level of 5-HTP to decline. Subsequently, the decreased 5-HTP levels further contributed to a lower concentration of 5-HT in the hippocampus. Eventually, the mice exhibited depressive-like behaviors [41]. In the present study, p-Firmicutes, f-Lactobacillaceae, and f-Ruminococcaceae were positively related to 5-HT levels, while p-Proteobacteria and g-Prevotella were negatively correlated with 5-HT levels. Proteobacteria and Actinobacteria, known as harmful bacteria, were found to be increased in CRS mice [29]. The supplementation of SCFA inhibited the conversion of tryptophan to kynurenine and restored the decrease in 5-HT levels in both the hippocampus and gut [42,43,44]. Mice that received oral supplementation of SCFAs showed alleviated stress-induced anxiety behaviors [45]. Our study has shown that propionic acid, valeric acid, and isobutyric acid were negatively correlated with tryptophan and tyrosine.

Furthermore, the alteration of acetic acid and propionic acid levels by differential genera within the *Firmicutes* phylum may lead to disturbances in peripheral and central GP metabolism [42]. Also, we found a significant upregulation of LCAT, PLA2G6, and PLA2G12A mRNA expression in the MD group as well as significantly increased LPC and LPE contents. As the enzyme responsible for extracellular lipoprotein metabolism, LCAT was synthesized mainly in the liver and secreted into the plasma and brain, where it converted PC into LPC [46]. PLA2G6 and PLA2G12, a secreted phospholipase A2, could deacylate PC and PE into LPC and LPE, respectively [47]. LPC and LPE were important signaling molecules involved in the regulation of inflammatory responses and enabled the release of proinflammatory cytokines [48]. Interestingly, mRNA expression of TNF-α, IL-6, and IL-1β in the brain was enhanced in MD mice. Finally, the validation experiment indicated that ZSSF could increase 5-HT and 5-HTP production and reduce LPC18:1(9Z) and PC 34:1 by inhibiting the activity of key enzymes involved in tryptophan and GP metabolism.

There were several limitations in our study. First, the levels of tryptophan and its derivatives were measured in the serum and brain but were not confirmed by Western blot or immunohistochemistry. Second, the validation of microbiota findings with FMT or antibiotics needs to be further explored. Further studies are essential to investigate the detailed host–microbe–food–nutrient interactions. The present study paved the way for future studies on the underlying molecular crosstalk mediated by ZSSF between the host and CRS microbiota.

## 5. Conclusions

In this study, experimental results demonstrated that ZSSF could reduce anxiety-like behaviors, exert sedative–hypnotic effects, increase hippocampal 5-HT and 5-HTP, and enhance intestinal barrier function by inhibiting colon ZO-1, Claudin-1, and Occludin expression and TNF-α, IL-6, and IL-1β levels in CRS mice. ZSSF could improve the gut microbiota composition with an increase in *Bacteroidetes* and a decrease in *Firmicutes* and an increase in fecal SCFAs, which might relate to the anti-anxiety effect. ZSSF upregulated the mRNA expression of TPH2 and DDC key enzymes in tryptophan metabolism and downregulated the relative expression of PLA2G12A, LACT, and PLA2G6 key enzymes in GP metabolism. Therefore, ZSSF has the potential to improve anxiety and sleep disorders as a natural functional food ingredient.

## Data Availability

The original contributions presented in this study are included in the article/Supplementary Materials, and further inquiries can be directed to the corresponding authors.

## Supplementary Materials

The following supporting information can be downloaded at: https://www.mdpi.com/article/10.3390/foods14050828/s1, Figure S1: ZSSFH regulated the gut microbiota composition. Relative abundance of gut microbiota at the family level. (A) Relative abundance of gut microbiota at the family level. (B) Relative quantitative analysis of major family (S24-7, Lactobacillaceae, Ruminococcaceae); Figure S2: Typical GC-MS chromatography of feces with standard mixture solutions and fecal sample; Figure S3. Effects of ZSSFH on the hypothalamus lipidomics and serum metabolism in CRS-induced anxiety-like mice. (A, C, E, G, I) positive ion mode, (B, D, F, H, J) negative ion mode. Hypothalamus lipidomics: (A and B) Coefficient of variation (CV) distribution of all detected lipids in QCs. (C and D) PCA score plot of four groups and QC. (E and F) The OPLS-DA score plot of CN and MD group. (G and H) Permutation test plot based on OPLS-DA methods. (I and J) The S+V plot on OPLS-DA method. (K) Hierarchical cluster analyses of differential metabolites in all groups. Red and blue of increasing intensity indicate the size of the relative expression of metabolite in groups of samples. Fold change (FC) (group MD versus group CN); (L) Pathway analysis of different metabolites among four groups. Table S1: The information of reference standards and ELISA kits; Table S2: The retention times (tR), declustering potentials (DPs), collision energies (CEs) of the seven chemical compounds; Table S3: The tR, qualitative ions and quantitative ion of SCFAs; Table S4: The detailed information of lipid internal standards (I.S.), lipid normalization and diagnostic fragments; Table S5: List of Real-Time qPCR primer; Table S6: Regression equation, correlation coefficient and linear range of seven components; Table S7: The regression equations, linear ranges and LOQs of SCFAs in mouse feces determined by GC-MS; Table S8: R2Y and Q2 of hypothalamus metabolism and lipidomics on OPLS-DA models; Table S9: Differential metabolites in positive and negative mode of hypothalamus metabolism; Table S10: Differential metabolites in positive and negative mode of hypothalamus lipidomics; Table S11: Differential metabolites in positive and negative mode of serum metabolism; Table S12: The number of r value > 0.9 in gut microbiota and SCFAs correlation analysis; Table S13: R value > 0.9 in SCFAs and serum metabolites correlation analysis.

## Abbreviations

5-HT, serotonin; Car, carnitine; Cer, ceramide; CORT, corticosterone; CRH, corticotropinreleasing hormone; CRS, chronic restraint stress; DG, diglycerides; DGLA, dihomo-gamma-linolenic acid; DHA, docosahexaenoic acid; DZP, diazepam; EPA, eicosapentaenoic acid; EPM, elevated plus maze; FA, fatty acid; GAD, generalized anxiety disorder; GPs, glycerophospholipids; GLs, glycerolipids; HPA, Hypothalamic-pituitary-adrenocortical; LPA, oleoyl-L-α-lysophosphatidic acid; LPC, lysophosphatidylcholine; OFT, open field test; OPLS-DA, orthogonal partial least squares discriminant analysis; PC, phosphatidylcholine; PCA, principal component analysis; PE, phosphatidyl ethanolamine; PG, phosphatidylglycerols; PLS-DA, partial least squares discrimination analysis; QC, quality control; SCFAs, short-chain fatty acids; SLs, sphingolipids; SM, sphingomyelin; ST, sleep test; ST, sterols; TCM, traditional Chinese medicine; TG, triacylglycerol; UPLC-Q-TOF-MS, ultra-performance liquid chromatography with a tandem quadrupole time-of-flight mass spectrometry; WGCNA, weighted correlation network analysis; ZSS, Ziziphi Spinosae Semen; ZSSF, Ziziphi Spinosae Semen flavonoid.
