# Supplementary material for: Ziziphi Spinosae Semen Flavonoid Ameliorates Hypothalamic Metabolism and Modulates Gut Microbiota in Chronic Restraint Stress-Induced Anxiety-like Behavior in Mice
Source: Foods. 2025 Feb 27;14(5):828. doi: 10.3390/foods14050828 (PMC11898499; doi:10.3390/foods14050828)
Supplement: Supplementary file 1 [file foods-14-00828-s001.zip › foods-3462769-supplementary.pdf]

Supplementary Flies

Materials and Methods

S1. UPLC-Q Trap/MS/MS analysis of seven compounds in ZSSF

S1.1 Reference solution preparation

Single reference stock solution (1 mg/mL) of spinosin and 6'''-feruloylspinosin, swertisin, vicenin II, kaempferol-3-*O*-rutinoside, isovitexin, rutin were prepared with 70% methanol, respectively. The mixed reference solution containing each of the above seven standards was prepared in the final concentration of 60 μg/mL, 30 μg/mL, 10 μg/mL, 5 μg/mL, 4 μg/mL, 1 μg/mL, and 2 μg/mL, respectively. The above mixed reference solution was diluted stepwise with 70% methanol into 7 concentration gradients of mixed reference solution.

S1.2 Chromatographic and mass spectrometry conditions

Qualitative analysis and quantitative analysis were performed by using an ACQUITY UPLC ®HSS T3 (2.1×150 mm, 1.8 μm). The gradient mobile phases were 0.1% formic acid in water (A) and 0.1% formic acid in acetonitrile (B) with the flow rate at 0.3 mL/min. The gradient elution was optimized as follows: 5%-17% B from 0 to 8 min, 17% B from 8 to 10 min, 17%-18% B from 10 to 11 min, 18%-20% B from 11 to 12 min, 20%-23% B from 12 to 17 min, 23%-33% B from 17 to 22 min, 33%-100% B from 22 to 25 min. The injection volume was 2 μL.

Mass spectrometry was performed using a Q-Trap 3200 system with a Duo Spray Ion source in the negative electrospray ion mode (AB SCIEX, Redwood, CA, USA) with the use of the following parameter settings: ion spray voltage -4500 V; ion source temperature of 550°C, a declustering potential (DP) of -60 V. Moreover, curtain gas, nebulizer gas (Gas 1), and heater gas (Gas 2) were set to 40, 50, and 50 psi, respectively. The IDA threshold was set to 500 counts per second (cps) for MRM scans, while the EPI scan rate was 1000 Da/s with dynamic fill in the trap and the scan range was from 50 to 950 Da. The collision energy (CE) was set at -40 ± 10 eV and the declustering potential (DP) was set at 60 eV. Entrance potential (EP) for analytes was -10 eV. Data was processed with PeakView® 2.2 software (AB SCIEX, Foster, CA, USA).

S2. Behavioral tests

Except the control group, the other groups were individually placed into a well-ventilated 50 mL conical tube, plugged with a middle tube 3 cm in length, and then the tube was capped. Mice were not able to move forward or backward in this device. Restraint stress was delivered to mice at set times daily from 10:00 AM to 1:00 PM for 3 h.

S2.1 Elevated plus maze (EPM)

On day 15 and 16 of the experiment, the EPM test was performed. The EPM consisted of two open arms (41 cm × 15 cm), two closed arms (41 cm × 15 cm × 15 cm), a central area (15 cm × 15 cm), and the apparatus was elevated 30 cm above the floor. with a digital camera positioned directly above. The mice were placed in the central area and the numbers of entries into the arms and the time spent in each arm were automatically recorded over a period of 5 min. The percentages of number of entries into the open arms and time spent in the open arms were calculated as follows:

Percentage of Entries $_{\text{into open arms}}$ = Entries $_{\text{into open arms}}$/ (Entries $_{\text{into open arms}}$ + Entries $_{\text{into closed arms}}$) × 100% and Percentage of Time $_{\text{spent in open arms}}$

= Time $_{\text{spent in open arms}}$/ (Time $_{\text{spent in open arms}}$ + Time $_{\text{spent in closed arms}}$) × 100%; respectively. Anxiety index=1-($\frac{open\ arm\ time}{total\ time\ on\ the\ maze}+\frac{open\ arm\ entry}{total\ entry}$)/2.

The anxiety index values range from 0 to 1, with a higher value indicating increased anxiety.

S2.2 Sleep test (ST)

One day 17 and 18 of the experiment schedules, 30 min after the gavage, the mice were intraperitoneal injection (i.p.) of pentobarbital sodium (46 mg/kg used as the hypnotic dose), mice were placed in individual cages for the test. The mice were placed on a black foam pad and abdomen up. For sleep latency, the time was recorded from pentobarbital injection to the time when righting reflex disappeared. For sleep duration, the time was recorded until the righting reflex recovered.

S2.3 Open field test (OFT)

On day 19 and 20 of the experiment schedule, the open field test (OFT) was performed in a white open box (50 cm × 50 cm × 50 cm) in which the arena was divided into 25 equal

squares. The mice were placed in the center area of the center box for 5 min. Behavioral parameters, such as total distance and rearing number were measured. Between trials, the maze was cleaned with 75% ethanol to remove odor cues.

S3. Histopathological and immunofluorescence analysis

The hippocampus and colon tissues were fixed in 4% paraformaldehyde solution, followed by embedding in paraffin. Subsequently, the tissue sections were subjected to staining using standard hematoxylin-eosin (H&E) and alcian blue-periodic acid-Schiff (AB-PAS) methods. The NIKON Eclipse Ci microscope (Eclipse-ci, Nikon, Japan) was employed for slide observation.

Paraffin-embedded colon tissues were sliced to 5 μm sections, which were deparaffinized in xylene and stained with primary antibodies against tight junction (TJ) proteins including anti-Claudin (1:400, Proteintech, Chain), anti-Occludin (1:400, Abcam, UK), anti-ZO-1 (1:400, Boster, Chain). Then, the corresponding primary antibodies were detected using the appropriate secondary antibody, goat anti-rabbit Alexa Fluor 488 (1:400, Servicebio, China) and the DAPI solution (Servicebio, China) was employed for nuclear detection. The representative images were captured by a laser confocal microscopy (Olympus, Japan). The quantification of positively stained cells was conducted using Image J software.

S4. Fecal SCFAs levels analysis

S4.1 GC-MS analysis

Quantitative and qualitative detection of SCFAs with Agilent 7890 B GC-MS coupled with an Agilent 5977 mass selective detector (Agilent, Santa Clara, CA, USA) and DB-FFAP capillary column (30 m×0.25 mm×0.25 μm), with helium as carrier gas at a constant flow rate of 1 mL/min. Samples (0.5 μL) were injected using a pressure pulsed split mode (10 psi) with a split ration of 10:1. The initial column oven temperature was 100 ºC for 1 min, and then increased to 110 ºC at a rate of 15 ºC/min and held for 0.67 min, increased to 120 ºC at a rate of 5 ºC/min and held for 1 min, increased to 130 ºC at a rate of 10 ºC/min, increased to 140 ºC at a rate of 3 ºC/min and held for 1 min then increased to a final temperature of 220 ºC at a rate of 30 ºC/min and held for 3 min. The total run time was 14.7 min and all the analytes

were detected in selected ion monitoring (SIM) mode. Qualitative ions and quantitative ion m/z for each analyte and their respective internal standards were chosen based on signal intensity. All analytes were identified using the NIST database, to which the obtained spectra were compared.

S4.2 Reference solution preparation

Single reference stock solution of acetic acid (1.258×10^4 μg/mL), propionic acid (2383.2 μg/mL), isobutyric acid (171 μg/mL), butyric acid (2892 μg/mL), isovaleric acid (111 μg/mL), valeric acid (281.1 μg/mL), and isocaproic acid (92.3 μg/mL) were prepared with methanol. The mixed reference solution containing each of the above seven standards was prepared in the final concentration of 1049 μg/mL, 198.6 μg/mL, 14.25 μg/mL, 241 μg/mL, 9.25 μg/mL, 23.475 μg/mL, and 7.692 μg/mL, respectively. The above mixed reference solution was diluted stepwise with methanol into 7 concentration gradients of mixed reference solution. The concentration of internal standard (IS) 2-ethybutyric acid was 4600 μg/mL.

S4.3 Linearity and range

Standard calibration curves were constructed by spiking 40 mg of blank mice feces same as the

sample operation with 100 μL mixed reference solution and 6 μL IS. Limit of quantitation (LOQ) was the lowest concentration of SCFA in a sample which can be quantitatively determined.

Linear range was defined as the concentration range where the calibration curve displays linearity with correlation coefficient $R^2 > 0.99$, indicating that these methods could be employed extensively for quantifying SCFAs in biological samples with a wide range of concentrations. The relative error (RE) of LOQ with ± 20%, other concentration points with ± 15%. These results revealed that the sensitivity of the novel analysis method was good.

S5. Lipidomics based on UPLC-Q-TOF-MS analysis

For lipidomics, a Waters BEH C_8 column (1.7 μm, 2.1 mm × 100 mm,) was used for lipid separation. The mobile phases consisted of 60:40 (v/v) ACN/H_2O (10 mM AcAm, phase A) and 90:10 (v/v) IPA/ACN (10 mM AcAm, phase B). The flow rate was set as 0.26 mL/min and the column temperature was 55 °C with 5 μL injections. The gradient elution employed

as follows: 0-1.5 min, 32% B; 1.5-15.5 min, 32%-85% B; 15.5-15.6 min, 85%-97% B; 15.6-18 min, 97% B. The ion spray voltage for MS was set at 5.5 kV/-4.5 kV, respectively. The interface heater temperature was 500 °C and 550 °C in positive and negative ion modes, respectively. GS1, GS2, and CUR were set at 55, 55, and 30 psi. The MS scan range was *m/z* 50-1500 Da.

The original data (.wiff) from the polar part sample were first converted into .abf file format using Analysis Base File Converter. Then the converted data were processed using MS-DIAL (version 4.8) for peak detection, deconvolution, peak alignment and peak filling to obtain the data matrix including compound retention time, m/z, peak intensity information, and MS/MS fragments. The metabolites were identified by searching in PubChem databases using MS and MS/MS information (https://pubchem.ncbi.nlm.nih.gov/), HMDB (http://www.hmdb) and OSI SMMS software (the Standards database, Dalian Chem Data Solution Information Technology Co., Ltd., PR China). The metabolites were identified through Lipid Maps (https://lipidmaps.org/) and Mass Bank (https://massbank.eu/MassBank/).

S6. Metabolomics based on UPLC-Q-TOF-MS analysis

Chromatographic separation was carried out on HSS T3 column (1.8 μm，2.1×150 mm, Waters Crop., Milford, USA) using 1290 Infinity UPLC system. The mobile phase comprised of 0.1% formic acid in water (A) and ACN (B). The gradient elution employed as follows: 0-1 min, 2% B; 1-3 min, 2%-50% B; 3-14 min, 50%-60% B; 14-18 min, 60%-90% B; 18-19 min, 90%-100% B; 19-20 min, 100% B. Mass spectrometry analysis was performed on the Q-TOF 5600^+ mass spectrometer (AB SCIEX, Redwood City, CA, USA) with electrospray ionization (ESI) in both positive and negative modes, respectively. The interface heater temperature was 450 °C. The MS scan range was m/z 50-1500. The operation parameters of MS were applied as follows: the curtain gas (CUR), ion source gas 1(GS1), ion source gas 2 (GS2), turbo spray temperature (TEM), declustering potential (DP), collision energy (CE), collision energy spread (CES) were set at 30 psi, 55 psi, 55 psi, 450 °C, 60 V, 35 V, 15 eV. The flow rate was 0.35 mL/min and the column temperature was controlled at 50 °C with 5 μL injections.

S7. Serum metabolomics

Chromatographic separation was carried out on ACQUITY UPLC® BEH C8 column

(1.7 μm，2.1×100 mm) and HSS T3 column (1.8 μm，2.1×150 mm, Waters Crop., Milford, USA) using 1290 Infinity UPLC system. The flow rate was 0.35 mL/min and the column temperature was controlled at 50 °C with 5 μL injections. The mobile phase in positive mode was composed of water (A) and acetonitrile (B) each containing 0.1% formic acid, and the gradient elution was optimized as follows: 0-1 min, 5% B; 1-24, 5%-100% B; 24-28 min, 100% B. The mobile phase in negative mode was composed of water (A) and 95% acetonitrile (B) each containing 6.5 mmol ammonium bicarbonate, and the gradient elution was optimized as follows: 0-1 min, 2% B; 1-18 min, 100% B; 18-22 min, 100% B. Mass spectrometry analysis was performed on the Q-TOF 5600^{+} mass spectrometer (AB SCIEX, Redwood City, CA, USA) with electrospray ionization (ESI) in both positive and negative modes, respectively. The spray voltages were set at 5.5 kV/-4.5 kV, respectively. The operation parameters of MS were applied as follows: the curtain gas (CUR), ion source gas 1(GS1), ion source gas 2 (GS2), turbo spray temperature (TEM), declustering potential (DP), collision energy (CE), collision energy spread (CES) were set at 30 psi, 55 psi, 55 psi, 450 °C, 60 V, 40 V, 20 eV. The scanning mode was full scan mode from *m/z* 100 to 1500.

The MS/MS spectra was the information-dependent acquirement technology (IDA). The IDA-based auto-MS2 was implemented on the top 10 most intense metabolite ions in a cycle of full scan (0.25 s). Dynamic background subtraction (DBS), and charge monitoring to exclude multiply charged ions and isotopes were invoked during the data acquisition.

Figure legends

Figure S1. ZSSFH regulated the gut microbiota composition. Relative abundance of gut microbiota at the family level. (A) Relative abundance of gut microbiota at the family level. (B) Relative quantitative analysis of major family (S24-7, Lactobacillaceae, Ruminococcaceae).

Figure S2. Typical GC-MS chromatography of feces with standard mixture solutions and fecal sample.

Figure S3. Effects of ZSSFH on the hypothalamus lipidomics and serum metabolism in CRS-induced anxiety-like mice. (A, C, E, G, I) positive ion mode, (B, D, F, H, J) negative ion mode. Hypothalamus lipidomics: (A and B) Coefficient of variation (CV) distribution of all detected lipids in QCs. (C and D) PCA score plot of four groups and QC. (E and F) The OPLS-DA score plot of CN and MD group. (G and H) Permutation test plot based on OPLS-DA methods. (I and J) The S+V plot on OPLS-DA method. (K) Hierarchical cluster analyses of differential metabolites in all groups. Red and blue of increasing intensity indicate the size of the relative expression of metabolite in groups of samples. Fold change (FC) (group MD versus group CN); (L) Pathway analysis of different metabolites among four groups.

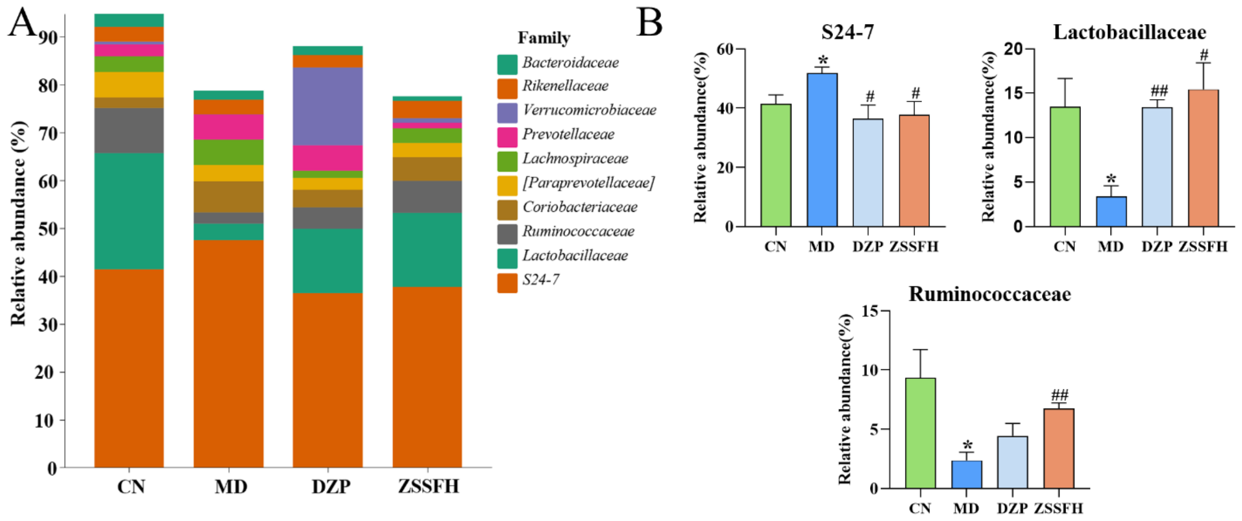

Figure S1. ZSSFH regulated the gut microbiota composition. Relative abundance of gut microbiota at the family level. (A) Relative abundance of gut microbiota at the family level. (B) Relative quantitative analysis of major family (S24-7, Lactobacillaceae, Ruminococcaceae).

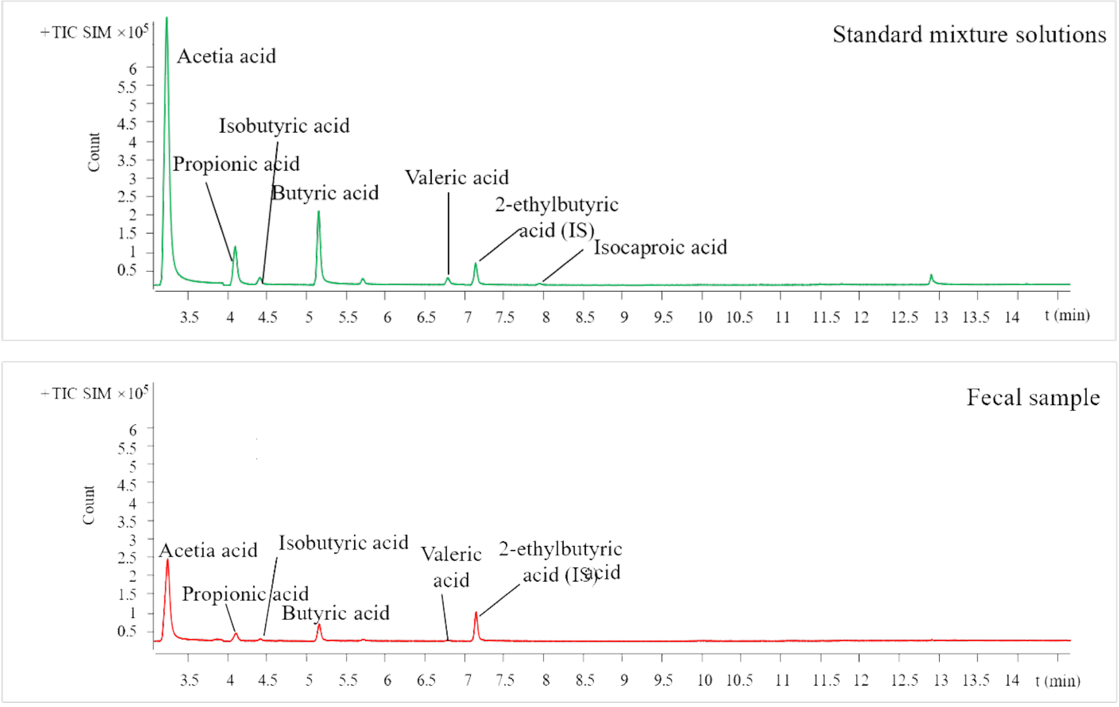

Figure S2. Typical GC-MS chromatography of feces with standard mixture solutions and fecal sample.

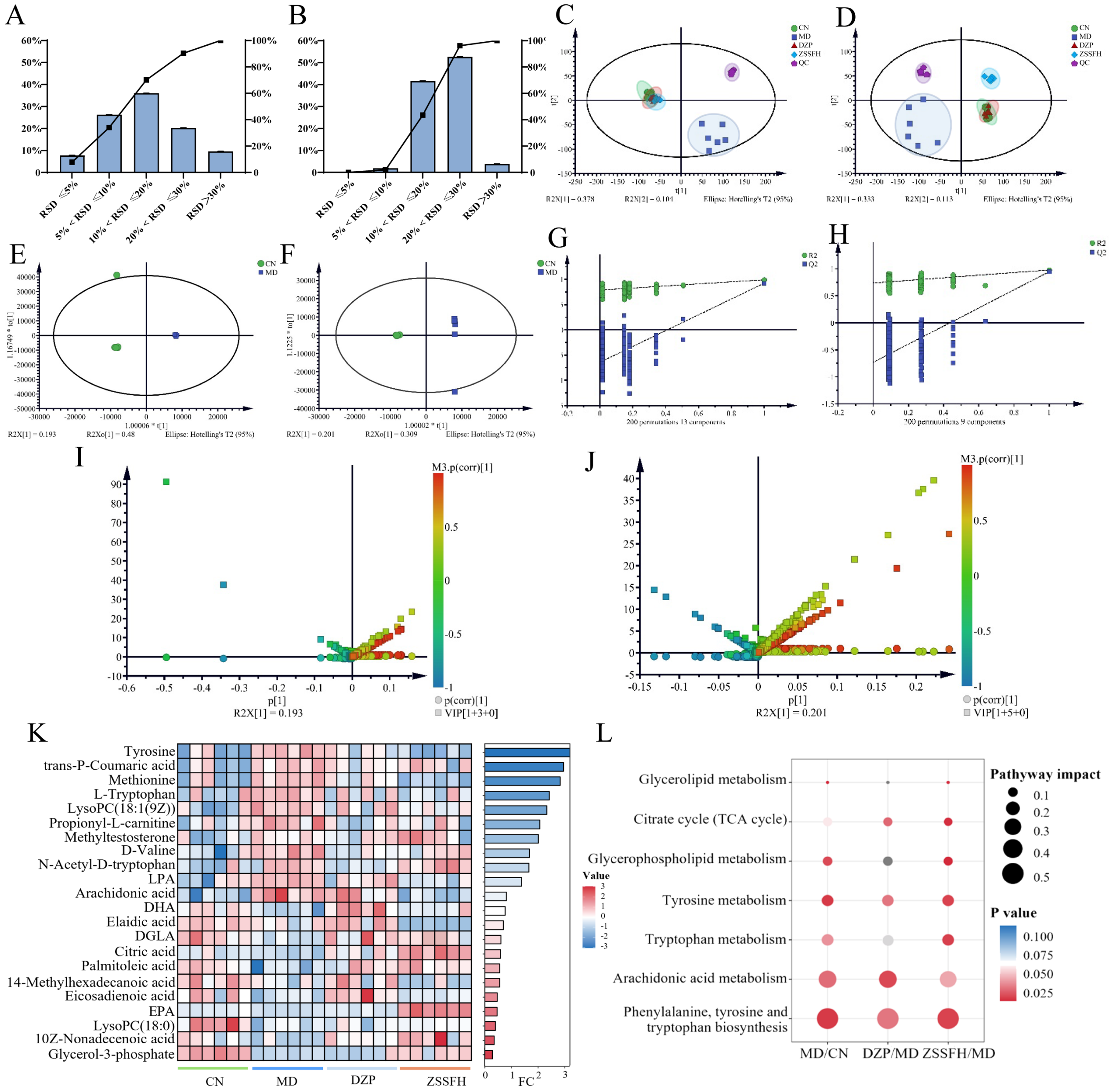

Figure S3. Effects of ZSSFH on the hypothalamus lipidomics and serum metabolism in CRS-induced anxiety-like mice. (A, C, E, G, I) positive ion mode, (B, D, F, H, J) negative ion mode. Hypothalamus lipidomics: (A and B) Coefficient of variation (CV) distribution of all detected lipids in QCs. (C and D) PCA score plot of four groups and QC. (E and F) The OPLS-DA score plot of CN and MD group. (G and H) Permutation test plot based on OPLS-DA methods. (I and J) The S+V plot on OPLS-DA method. (K) Hierarchical cluster analyses of differential metabolites in all groups. Red and blue of increasing intensity indicate the size of the relative expression of metabolite in groups of samples. Fold change (FC) (group MD versus group CN); (L) Pathway analysis of different metabolites among four groups.

Table Captions

Table S1. The information of reference standards and ELISA kits.

Table S2. The retention times (t_R), declustering potentials (DPs), collision energies (CEs) of the seven chemical compounds.

Table S3. The t_R, qualitative ions and quantitative ion of SCFAs.

Table S4. The detailed information of lipid internal standards (I.S.), lipid normalization and diagnostic fragments.

Table S5. List of Real-Time qPCR primer.

Table S6. Regression equation, correlation coefficient and linear range of seven components.

Table S7. The regression equations, linear ranges and LOQs of SCFAs in mouse feces determined by GC-MS.

Table S8. R^2Y and Q^2 of hypothalamus metabolism and lipidomics on OPLS-DA models.

Table S9. Differential metabolites in positive and negative mode of hypothalamus metabolism.

Table S10. Differential metabolites in positive and negative mode of hypothalamus lipidomics.

Table S11. Differential metabolites in positive and negative mode of serum metabolism.

Table S12. The number of r value > 0.9 in gut microbiota and SCFAs correlation analysis.

Table S13. R value > 0.9 in SCFAs and serum metabolites correlation analysis.

Table S1. The information of reference standards and ELISA kits.

Compounds and reagents	Company	Regions
swertisin	Baoji Herbest Bio-Tech Co., Ltd.	Baoji, China
spinosin	Baoji Herbest Bio-Tech Co., Ltd.	Baoji, China
6‴-feruloylspinosin	Baoji Herbest Bio-Tech Co., Ltd.	Baoji, China
rutin	Baoji Herbest Bio-Tech Co., Ltd.	Baoji, China
Kaempferol 3-*O*-rutinoside	Shanghai Standard technology Co., Ltd.	Shanghai, China
vicenin II	Shanghai Standard technology Co., Ltd.	Shanghai, China
isovitexin	Shanghai Standard technology Co., Ltd.	Shanghai, China
corticosterone (CORT), corticotropin-releasing hormone (CRH), serotonin (5-HT),	Beijing Jimei Biotechnology Co., Ltd.	Beijing, China
5-hydroxytryptophan (5-HTP)	Jianglai industrial Limited by Share Ltd.	Shanghai, China
tumor necrosis factor-α (TNF-α), interleukin (IL)-6, IL-1β	Bioswamp Biotechnology Co., Ltd.	Wuhan, China
SCFAs standards	Tokyo Chemical Industry.	Tokyo, Japan
phosphatidylcholine (PC), phosphatidyl ethanolamine (PE), lysophosphatidylcholine (LPC), ceramide (Cer)	Avanti Polar Lipids.	Milan, Italy
triacylglycerol (TG)	Cayman Chemical.	Milan, Italy
free fatty acid (FFA)	Toronto Research Chemicals Inc.	Liverpool, UK

Table S2. The retention times (t_R), declustering potentials (DPs), collision energies (CEs) of the seven chemical compounds.

Compounds	RT (min)	Ion transition Precursor > production	DP (V)	CE (eV)
swertisin	14.18	445.3 > 297.3	-75	-45
vicenin II	9.33	593.5 > 353.4	-90	-47
kaempferol-3-*O*-rutinoside	15.48	593.5 > 284.2	-86	-49
spinosin	13.28	607.5 > 292.4	-90	-63
isovitexin	13.59	431.2 > 283.2	-57	-46
6‴-feruloylspinosin	17.78	783.7 > 427.3	-140	-48
rutin	13.34	609.4 > 300.3	-100	-50

Table S3. The t_R, qualitative ions and quantitative ion of SCFAs.

Analyte	RT (min)	Qualitative ions	Quantitative ion
Acetic acid	3.257	43, 60	45
Propionic acid	4.122	57, 73	74
Isobutyric acid	4.431	41, 73	43
Butyric acid	5.176	41, 73	60
Valeric acid	6.809	41	60

Table S4. The detailed information of lipid internal standards (I.S.), lipid normalization and diagnostic fragments.

I.S.	Concentration(μg/mL)	*m*/*z*(+)	*m*/*z*(-)	Normalized lipid classes	Diagnostic fragments
PC 19:0/19:0	1.6	$[M+H]^+$ 818.6630	$[M+HCOO]^-$ 860.6338	PC, PG, PS, PI	$[M+H]^+$ 184.0733<br>$[M+CH_3COO]^-$ 74.0368
LPC 19:0	0.8	$[M+H]^+$ 538.388	$[M+CH_3COO]^-$ 596.3915	LPC, LPI	$[M+H]^+$ 184.0733
PE 17:0/17:0	0.8	$[M+H]^+$ 720.5372	/	LPE, LPE O, PE, PE O	$[M+H]^+$ 141.0191<br>$[M-H]^-$ 140.0118, 196.0380
TG 15:0/15:0/15:0	1.25	$[M+NH_4]^+$ 782.7232	/	DG, MG, TG	$FA+NH_3$
Cer d18:1/17:0	0.5	$[M+H]^+$ 552.5341	/	CE, Cer, HexCer	$[M+H]^+$ 264.2685
FFA 16:0_d3	0.8	/	$[M-H]^-$ 286.2876	FFA	/

Table S5. List of Real-Time qPCR primer.

Gene	Sequence
TPH2	Forward: 5′-GAGCGACAAGACAGCGGTAGTG-3′<br>Reverse: 5′-TTCGTCGGGACCTCCTGGATTC-3′
DDC	Forward: 5′-CTGCTCTGCCATGTGGGTGAAG-3′<br>Reverse: 5′-GCCAGTGCCTGTAGTCAGTGATG-3′
LCAT	Forward: 5′-CAACGGATATGTGCGGGATGAGAC-3′<br>Reverse: 5′-AGCGGCATACATCTCCTCTACCAG-3′
PLA2G12A	Forward: 5′-CTCCTCCTCCTCCTGCTGCTG-3′<br>Reverse: 5′-TGTCTATCTTGTGGATGCCGTTGC-3′
PLA2G6	Forward: 5′-CGTCCTGCTGCTCTGTAATGCC-3′<br>Reverse: 5′-TTGCTGTGGATCTGGTTGCTGTC-3′
TNF-α	Forward: 5′-ACGCTCTTCTGTCTACTGAACTTCG-3′<br>Reverse: 5′-TGGTTTGTGAGTGTGAGGGTCTG-3′
IL-6	Forward: 5′-TGGGACTGATGCTGGTGACAAC-3′<br>Reverse: 5′-GTGGTATCCTCTGTGAAGTCTCCTC-3′
IL-1β	Forward: 5′-CTCGCAGCAGCACTACAACAAG-3′<br>Reverse: 5′-CCACGGGAAAGACACAGGTAG-3′
GAPDH	Forward: 5′-AACTTTGGCATTGTGGAAGGGCTC-3′<br>Reverse: 5′-TGGAAGAGTGGGAGTTGCTGTTGA-3′

Table S6. Regression equation, correlation coefficient and linear range of seven components.

Analyte	Regression equation	*r*	Linea rage (μg/mL)
swertisin	$Y = 1.29 \times 10^5 X - 1.82 \times 10^3$	0.9995	0.025-1
vicenin II	$Y = 4.51 \times 10^4 X - 4.68 \times 10^3$	0.9995	0.125-5
kaempferol-3-*O*-rutinoside	$Y = 1.08 \times 10^5 X - 7.40 \times 10^3$	0.9995	0.04-4
spinosin	$Y = 2.45 \times 10^4 X - 9.81 \times 10^3$	0.9996	1-60
isovitexin	$Y = 6.44 \times 10^4 X - 1.02 \times 10^3$	0.9995	0.05-1
6‴-feruloylspinosin	$Y = 2.94 \times 10^4 X - 9.81 \times 10^3$	0.9997	1.5-30
rutin	$Y = 1.20 \times 10^5 X - 5.30 \times 10^3$	0.9995	0.05-2

Table S7. The regression equations, linear ranges and LOQs of SCFAs in mouse feces determined by GC-MS.

Analyte	Regression equation	R^2	LOQ (μg/g)	Linea rage (μg/g)
Acetic acid	Y = 0.0192X-0.7908	0.9909	0.86	0.86-4.92
Propionic acid	Y = 0.0165X-0.1055	0.9920	0.17	0.17-8.13
Isobutyric acid	Y = 0.0472X-0.0339	0.9972	0.029	0.029-0.52
Butyric acid	Y = 0.0374X-0.1413	0.9954	0.49	0.49-9.86
Valeric acid	Y = 0.0538X-0.0377	0.9939	0.048	0.048-0.864

Table S8. R^2Y and Q^2 of hypothalamus metabolism and lipidomics on OPLS-DA models.

Model	Acquisition	R^2Y	Q^2
Hypothalamus	Positive	0.991	0.965
metabolomics	Negative	0.998	0.963
Hypothalamus	Positive	0.989	0.901
lipidomics	Negative	0.996	0.936

Table S9. Differential metabolites in positive and negative mode of hypothalamus metabolism.

NO.	Metabolites	Formular	Ion mode	m/z	error	RT (min)	MS fragments (m/z)	MD	DZP	ZSSFH	HMDB ID	KEGG
1	Spermine	$C_{10}H_{26}N_4$	$[M+H]^+$	203.2209	0.2	0.91	129.1388, 112.1123, 84.0814, 72.0814, 70.0662, 58.0675	↓*	↓	↑#	HMDB 0001256	C00750
2	L-Arginine	$C_6H_{14}N_4O_2$	$[M+H]^+$	175.1178	6.5	1.15	71.0500, 70.0661, 60.0583	↓*	↓	↑#	HMDB 0000157	C00062
3	Choline	$C_5H_{13}NO$	$[M+H]^+$	104.1067	2.0	1.18	69.0349, 60.0821, 59.0746, 58.0670, 56.0504	↑***	↓#	↓	HMDB 0000097	C00114
4	Carnitine	$C_7H_{15}NO_3$	$[M+H]^+$	162.1113	-6.7	1.22	103.0398, 102.0925, 85.0299, 60.0839, 59.0759, 58.0686	↑**	↓#	↓#	HMDB 00000062	C00318
5	L-Acetylcarnitine	$C_9H_{17}NO_4$	$[M+H]^+$	204.1215	4.3	1.85	188.1009, 85.0296, 84.0824,	↑***	↓##	↓###	HMDB 0000201	C02571
6	Methionine	$C_5H_{11}O_2NS$	$[M+H]^+$	150.0571	-3.6	1.88	102.0543, 87.0279, 74.0257, 61.0136	↑**	↓###	↓###	HMDB 0000696	C00855
7	Hypoxanthine	$C_5H_4N_4O$	$[M+H]^+$	137.0457	7.9	1.94	120.0184, 119.0342, 110.0352, 94.0400, 93.0070, 92.0243, 83.0238, 82.0392, 67.0287, 65.0142, 55.0316	↑**	↓#	↓	HMDB 0000157	C00262
8	Xanthine	$C_5H_4N_4O_2$	$[M+H]^+$	153.0393	9.0	2.05	136.0132, 135.0309, 110.0351, 108.0195, 93.0069, 83.0250, 82.0405, 81.0085, 55.0316	↑***	↓##	↓##	HMDB 0000292	C00385
9	Tyrosine	$C_9H_{11}NO_3$	$[M+H]^+$	182.0796	8.1	2.47	136.0757, 123.0442, 119.0493, 107.0492, 77.0397	↑**	↓	↓#	HMDB 00000158	C00082
10	Glutamic acid	$C_5H_9NO_4$	$[M+H]^+$	148.0593	-1.1	2.63	102.0566, 84.0451, 70.0409, 56.0595	↑**	↓	↓###	HMDB 0000148	C00025
11	L-Norleucine	$C_6H_{13}NO_2$	$[M+H]^+$	132.1020	5.1	2.97	86.0964, 69.0699, 58.0651, 56.0495, 44.0495, 43.0542	↑**	↓#	↓#	HMDB 0001645	C01933

12	LysoPC(18:1(9Z))	$C_{26}H_{52}NO_7P$	$[M+H]^+$	522.3529	3.6	14.33	505.3420, 184.0729, 166.0612, 125.0001, 86.0982, 60.0846	$\uparrow^{***}$	$\downarrow^{\#\#\#}$	$\downarrow^{\#\#\#}$	HMDB 0002815	C04230

**NO.**	**Metabolites**	**Formular**	**Ion mode**	**m/z**	**error**	**RT (min)**	**MS fragments (m/z)**	**MD**	**DZP**	**ZSSFH**	**HMDB ID**	**KEGG**
13	LysoPC(18:0)	$C_{26}H_{54}NO_7P$	$[M+H]^+$	524.36	3.6	17.48	506.3582, 447.2882, 341.3050, 258.1111, 184.7588, 184.6322, 184.4252, 184.0727, 166.0628, 163.0146, 124.9997, 104.1078, 86.0977, 71.0758, 60.0841	$\uparrow^{*}$	$\downarrow$	$\downarrow$	HMDB 0010384	C04230
14	N-Acetylaspartate	$C_6H_9NO_5$	$[M-H]^-$	174.0413	7.3	1.89	137.9381, 88.0437, 58.0312, 41.9984	$\uparrow^{*}$	$\downarrow^{\#}$	$\downarrow$	HMDB 0000812	C01042
15	Isocitric acid	$C_6H_8O_7$	$[M-H]^-$	191.0191	-3.4	3.14	111.0098	$\uparrow^{**}$	$\downarrow$	$\downarrow^{\#}$	HMDB 0000193	C00311
16	Inosine	$C_{10}H_{12}N_4O_5$	$[M-H]^-$	267.0737	3.7	3.18	135.0312,108.0211	$\uparrow^{***}$	$\downarrow$	$\downarrow^{\#\#\#}$	HMDB 0000195	C00294
17	Phenylalanine	$C_9H_{11}NO_2$	$[M-H]^-$	164.0721	8.1	3.40	147.0450, 118.0671, 103.0562, 91.0547, 79.0551, 77.0396, 51.0268	$\uparrow^{***}$	$\downarrow$	$\downarrow^{\#\#}$	HMDB 0000159	C00079
18	Tryptophan	$C_{11}H_{12}N_2O_2$	$[M-H]^-$	203.0817	1.7	3.60	186.0540, 142.0663, 117.0538, 116.0507	$\downarrow^{**}$	$\downarrow$	$\uparrow^{\#}$	HMDB 0000929	C00078
19	Palmitic acid	$C_{16}H_{32}O_2$	$[M-H]^-$	255.2310	4.0	4.03	237.2252	$\uparrow^{**}$	$\downarrow$	$\downarrow^{\#\#}$	HMDB 0000220	C00249
20	Azelaic acid	$C_9H_{16}O_4$	$[M-H]^-$	187.0971	-2.9	4.4	125.0997,123.0828	$\uparrow^{***}$	$\downarrow^{\#\#\#}$	$\downarrow^{\#\#\#}$	HMDB 0000784	C08261
21	Uridine	$C_9H_{12}N_2O_6$	$[M-H]^-$	243.0611	4.4	4.44	152.0327, 122.0255, 111.0164, 110.0239	$\uparrow^{**}$	$\downarrow$	$\downarrow$	HMDB 0000296	C00299
22	11b-PGF2a	$C_{20}H_{34}O_5$	$[M-H]^-$	353.23312	1.1	5.19	129.0599	$\uparrow^{***}$	$\downarrow$	$\uparrow$	HMDB 0010199	C05959

23	Arachidonic acid	$C_{20}H_{32}O_2$	$[M-H]^-$	303.2318	1.4	19.88	259.2430	↑**	↓	↓#	HMDB 0001043	C00219
24	trans-Vaccenic acid	$C_{18}H_{34}O_2$	$[M-H]^-$	281.2470	4.9	20.79	59.0139, 44.9982, 41.0033	↑**	↓	↓#	HMDB 0003231	C08267

NO.	Metabolites	Formular	Ion mode	m/z	error	RT (min)	MS fragments (m/z)	MD	DZP	ZSSFH	HMDB ID	KEGG
25	Stearic acid	$C_{18}H_{36}O_2$	$[M-H]^-$	283.2645	-0.1	20.85	265.2538	↑*	↓	↓#	HMDB 0000827	C01530

Note: ↑ means the abundance of metabolites was increased, while ↓ means the abundance of metabolites was decreased. * means MD group compared with CN group and # means DZP and ZSSFH groups compared with MD group. Data were expressed as mean ± SEM; n = 6. (*, # $p < 0.05$, **, ## $p < 0.01$, ***, ### $p < 0.001$)

Table S10. Differential metabolites in positive and negative mode of hypothalamus lipidomics.

NO.	Metabolites	Formular	Ion mode	m/z	error	RT (min)	MS fragments (m/z)	MD	DZP	ZSSFH	HMDB ID	LIPID MAPS/ KEGG
1	LPC 18:2/0:0	$C_{26}H_{50}NO_7P$	$[M+H]^+$	520.3325	9.8	1.10	184.0775	↑*	↓	↓#	HMDB 0010386	LMGP0 1050035
2	LPC 16:0/0:0	$C_{24}H_{50}NO_7P$	$[M+H]^+$	496.3352	9.2	2.17	184.0718	↑*	↓	↓#	HMDB 0256091	LMGP0 1050113
3	LPE 20:1	$C_{25}H_{50}NO_7P$	$[M+H]^+$	508.3394	0.7	2.93	184.0698	↓*	↑	↓	HMDB 0011482	LMGP0 2050046
4	PS 44:12丨PS 22:6_22:6	$C_{50}H_{74}NO_{10}P$	$[M+H]^+$	880.5100	2.6	5.54	184.0755	↑***	↓#	↓###	HMDB 0012450	LMGP0 3010985
5	ST 27:2;O	$C_{27}H_{44}O$	$[M+Na]^+$	407.3270	3.2	6.09	325.2890	↓	↑	↓	HMDB 0000032	LMST0 1010069
6	PS 40:7丨PS 18:1_22:6	$C_{46}H_{76}NO_{10}P$	$[M+H]^+$	834.5285	-0.6	6.67	184.0641	↑***	↓	↓	HMDB 0012445	LMGP0 3010828
7	PS 40:6	$C_{46}H_{78}NO_{10}P$	$[M+Na]^+$	858.5194	7.2	7.52	651.5205, 184.0697	↑***	↓	↓###	HMDB 0112438	LMGP0 3010365
8	PS 40:6丨PS 18:0_22:6	$C_{46}H_{78}NO_{10}P$	$[M+H]^+$	836.5432	0.4	7.54	184.0695	↑***	↓##	↓###	HMDB 0010167	LMGP0 3010040
9	PG 40:6丨PG 18:0_22:6	$C_{46}H_{79}O_{10}P$	$[M+NH_4]^+$	840.5689	7.1	7.58	183.1132	↑***	↓	↓##	HMDB 0010614	LMGP0 4010040
10	PG 34:1丨PG 16:0_18:1	$C_{40}H_{77}O_{10}P$	$[M+NH_4]^+$	766.5512	9.7	7.68	184.0677	↑***	↓	↓###	HMDB 0244000	/
11	PI 38:4	$C_{47}H_{83}O_{13}P$	$[M+NH_4]^+$	904.5826	9.2	7.73	184.0852	↑***	↑	↓	/	/
12	PS 36:2丨PS 18:1_18:1	$C_{42}H_{78}NO_{10}P$	$[M+H]^+$	788.5410	3.2	7.79	184.0701	↑***	↑	↓##	HMDB 0012390	LMGP0 3010030
13	PS 38:4丨PS 18:0_20:4	$C_{44}H_{78}NO_{10}P$	$[M+H]^+$	812.5365	8.7	7.81	184.0696	↑***	↓	↓###	HMDB 0012383	LMGP0 3010039

NO.	Metabolites	Formular	Ion mode	m/z	error	RT (min)	MS fragments (m/z)	MD	DZP	ZSSFH	HMDB ID	LIPID MAPS/ KEGG
14	PS 40:4	PS 18:0_22:4	$C_{46}H_{82}NO_{10}P$	$[M+H]^+$	840.5894	-7.3	8.31	184.0705	↑***	↓	↓#	HMDB 0112382	LMGP0 3010883
15	SM 42:3;2O	SM 18:1;2O/24:2	$C_{47}H_{91}N_2O_6P$	$[M+H]^+$	811.6502	2.9	8.99	628.6458	↓*	↑#	↑###	HMDB 0240615	/
16	Cer(d18:1/24:2)	$C_{47}H_{83}O_{13}P$	$[M+H]^+$	810.6677	7.2	9.10	282.2778	↑***	↑	↓#	HMDB 0240679	/
17	DG 38:4	DG 18:0_20:4	$C_{41}H_{72}O_5$	$[M+NH_4]^+$	662.5713	0.6	10.25	341.3028, 287.2359	↓*	↑##	↑###	HMDB 0007170	LMGL0 2010111
18	DG 38:4	$C_{41}H_{72}O_5$	$[M+Na]^+$	667.5264	1.1	10.25	361.2694	↓***	↑	↑###	HMDB 0007254	LMGL0 2010109
19	DG 36:2	DG 18:0_18:2	$C_{39}H_{72}O_5$	$[M+NH_4]^+$	638.5750	-5.2	10.30	341.2998, 337.2735	↓***	↑	↑#	HMDB 0007161	LMGL0 2010050
20	DG 40:4	$C_{43}H_{76}O_5$	$[M+Na]^+$	695.5529	8.0	10.72	363.2843	↓**	↑	↑##	HMDB 0007373	LMGL0 2010165
21	DG 38:2	$C_{41}H_{76}O_5$	$[M+Na]^+$	671.5621	-5.5	10.95	367.3213, 339.2829	↓**	↑	↑	HMDB 0007224	LMGL0 2010090
22	DG 42:2	$C_{45}H_{84}O_5$	$[M+Na]^+$	727.6222	-1.6	12.13	369.3351	↓**	↑	↑#	HMDB 0007378	LMGL0 2010231
23	TG 54:7	TG 18:2_18:2_18:3	$C_{57}H_{96}O_6$	$[M+NH_4]^+$	894.7478	7.5	12.69	599.4970	↓***	↑	↑##	/	LMGL0 3013037
24	TG 54:6	TG 18:2_18:2_18:2	$C_{57}H_{98}O_6$	$[M+NH_4]^+$	896.7685	1.8	13.06	599.4966	↓***	↑#	↑##	HMDB 0005474	LMGL0 3010371
25		$C_{55}H_{98}O_6$		872.7644	6.6	13.36	599.4966	↓**	↑#	↑#		

	TG 52:4丨TG 16:0_18:2_18:2		$[M+NH_4]^+$									HMDB 05390	LMGL03010141

NO.	Metabolites	Formular	Ion mode	m/z	error	RT (min)	MS fragments (m/z)	MD	DZP	ZSSFH	HMDB ID	LIPID MAPS/ KEGG
26	TG 48:2丨TG 16:0_16:1_16:1	$C_{51}H_{94}O_6$	$[M+NH_4]^+$	820.7354	4.2	13.39	237.2198	↓***	↑	↑###	HMDB 0005376	LMGL03010018
27	TG 54:5丨TG 18:1_18:2_18:2	$C_{57}H_{100}O_6$	$[M+NH_4]^+$	898.7832	2.9	13.46	599.4927	↓***	↑#	↑##	HMDB 05461	LMGL03010327
28	TG 52:3丨TG 16:0_18:1_18:2	$C_{55}H_{100}O_6$	$[M+Na]^+$	879.7359	6.0	13.74	601.5190, 577.5059, 575.5035	↓***	↑#	↑	HMDB 05384	LMGL03010121
29	TG 54:4丨TG 18:1_18:1_18:2	$C_{57}H_{102}O_6$	$[M+NH_4]^+$	900.7970	4.9	13.77	599.4961	↓***	↑#	↑#	HMDB 05455	LMGL03010288
30	PC 34:1	$C_{42}H_{82}NO_8P$	$[M+H]^+$	760.5803	6.2	20.54	184.0698	↑***	↓##	↓###	HMDB 0243985	/
31	FA 10:0	$C_{10}H_{20}O_2$	$[M-H]^-$	171.1398	-4.4	1.44	73.0334	↑***	↑	↓#	HMDB 0000511	LMFA01010010
32	FA 18:3; O	$C_{18}H_{30}O_3$	$[M-H]^-$	293.2131	-3.2	1.44	59.0167	↑*	↑	↓	/	LMFA02000157
33	FA 16:1; O	$C_{16}H_{30}O_3$	$[M-H]^-$	269.2116	2.0	1.47	251.1967, 225.2212	↑**	↑	↓#	/	SID387452756
34	FA 18:2; O	$C_{18}H_{32}O_3$	$[M-H]^-$	295.2272	2.4	1.55	277.2123, 251.2294	↑*	↑	↓#	/	LMFA02000227
35	FA 18:1; O	$C_{18}H_{34}O_3$	$[M-H]^-$	297.2437	-0.8	1.57	279.2333, 253.2522	↑*	↑	↓	/	LMFA02000203
36	FA 20:2	$C_{20}H_{36}O_2$	$[M-H]^-$	307.2636	2.1	2.84	289.2598	↑*	↑	↓#	HMDB 0244372	C16525
37	FA 22:1; 2 O	$C_{22}H_{42}O_4$	$[M-H]^-$	369.2991	5.1	3.07	351.3050	↑***	↑	↓###	/	/

NO.	Metabolites	Formular	Ion mode	m/z	error	RT (min)	MS fragments (m/z)	MD	DZP	ZSSFH	HMDB ID	LIPID MAPS/ KEGG
38	FA 18:1	$C_{18}H_{34}O_2$	$[M-H]^-$	281.2478	2.5	6.32	59.0139	↑	↑	↓	HMDB 0062703	/
39	PG 42:8 丨PG 20:4_22:4	$C_{48}H_{79}O_{10}P$	$[M-H]^-$	845.5382	-5.1	6.32	152.9959	↑***	↓#	↓	/	LMGP0 4010647
40	PG 38:5 丨PG 18:1_20:4	$C_{44}H_{77}O_{10}P$	$[M-H]^-$	795.5169	1.7	6.38	152.9927	↑*	↑	↓	HMDB 0010640	LMGP0 4010879
41	PI 38:4 丨PI 18:0_20:4	$C_{47}H_{83}O_{13}P$	$[M-H]^-$	885.5489	1.1	7.63	259.0228, 241.0111, 152.9965	↑***	↓	↓	HMDB 0009815	LMGP0 6010010
42	FA 26:0	$C_{26}H_{52}O_2$	$[M-H]^-$	395.3884	2.8	8.20	351.3991	↑**	↑	↓#	HMDB 0002356	LMFA0 1010026
43	PE O-40:6 丨PE O-18:0_22:6	$C_{45}H_{80}NO_7P$	$[M-H]^-$	776.5544	7.2	8.46	140.0154	↑**	↓	↓	HMDB 0011394	LMGP0 2030005
44	PC O-41:4 丨PC O-19:0_22:4	$C_{49}H_{92}NO_7P$	$[M+HCOO]^-$	882.6668	-8.6	9.86	73.0000	↑*	↓	↓#	/	/

Note: ↑ means the abundance of metabolites was increased, while ↓ means the abundance of metabolites was decreased. * means MD group compared with CN group and # means DZP and ZSSFH groups compared with MD group. Data were expressed as mean ± SEM; n = 6. (*, # $p < 0.05$, **, ## $p < 0.01$, ***, ### $p < 0.001$)

Table S11. Differential metabolites in positive and negative mode of serum metabolism.

NO.	Metabolites	Formular	Ion mode	m/z	error	RT (min)	MS fragments (m/z)	MD	DZP	ZSSFH	HMDB ID	KEGG
1	D-Valine	$C_5H_{11}NO_2$	$[M+H]^+$	118.0858	-3.4	0.86	72.0808, 59.0774, 57.0600, 56.0532, 55.0571, 53.0462	↑**	↓##	↓	HMDB 0250806	C06417
2	Methionine	$C_5H_{11}NO_2S$	$[M+H]^+$	150.0578	-2.9	1.26	133.0320, 104.0543, 102.0545, 87.0309, 74.0298, 61.0128, 56.0530	↑**	↓#	↓#	HMDB 0000696	C00855
3	trans-P-Coumaric acid	$C_9H_8O_3$	$[M+H]^+$	165.0532	4.3	1.36	123.0443, 119.0497, 95.0496, 91.0586, 77.0418, 65.0385	↑**	↓#	↓	HMDB 0002035	C00811
4	Tyrosine	$C_9H_{11}NO_3$	$[M+H]^+$	182.0797	3.7	1.36	165.0528, 147.0463, 136.0746, 123.0443, 121.0639, 119.0480, 118.0678, 109.0608, 107.0493, 103.0532, 95.0493, 91.0559, 90.7774, 77.0405, 65.0419	↑**	↓##	↓#	HMDB 0000158	C00082
5	Propionyl-L-carnitine	$C_{10}H_{19}NO_4$	$[M+H]^+$	218.1391	1.9	1.52	159.0618, 144.1023, 85.0294, 84.0822, 60.0826, 57.03875	↑*	↓#	↓	HMDB 0000824	C03017
6	Tryptophan	$C_{11}H_{12}N_2O_2$	$[M+H]^+$	205.0971	0.1	2.87	188.0709, 170.0583, 160.0708, 159.0929, 146.0595, 144.0811, 143.0722, 142.0663, 132.0802, 130.0646, 128.0511, 127.0519, 118.0653, 117.0577, 115.0541, 91.0558	↑*	↓	↓#	HMDB 0000929	C00078
7	LysoPC(18:1(9Z))	$C_{26}H_{52}NO_7P$	$[M+H]^+$	522.3571	-4.3	15.44	505.3420, 184.0729, 166.0612, 125.0001, 86.0982, 60.0846	↑**	↓	↓##	HMDB 0002815	/
8	Methyltestosterone	$C_{20}H_{30}O_2$	$[M+H]^+$	303.2332	4.4	15.71	285.2267, 201.1259, 197.1333, 187.1102, 183.1128, 175.1488, 173.1310, 171.1178, 169.0990, 161.1373, 161.1317, 169.1174, 157.1019, 155.0850, 147.1150, 145.0990, 143.0867, 141.0716, 133.1005, 131.0862, 129.0696, 128.0600, 121.1027, 119.0870, 117.0707, 115.0545, 107.0833, 105.0704, 95.0871, 93.0710, 91.0550, 81.0706, 79.0542, 77.0396, 69.0724, 67.0561	↑*	↓	↑	HMDB 0015655	C07198

NO.	Metabolites	Formular	Ion mode	m/z	error	RT (min)	MS fragments (m/z)	MD	DZP	ZSSFH	HMDB ID	KEGG
9	LysoPC(18:0)	C26H52NO7P	[M+H]+	524.3716	-2.1	16.36	506.3582, 447.2882, 341.3050, 258.1111, 184.7588, 184.6322, 184.4252, 184.0727, 166.0628, 163.0146, 124.9997, 104.1078, 86.0977, 71.0758, 60.0841	↓**	↑	↑	HMDB 0010384	/
10	Palmitoleic acid	$C_{16}H_{30}O_2$	[M+H]$^+$	255.2316	-0.7	17.94	130.0855	↓*	↑#	↑##	HMDB 0003229	C08362
11	Dihomo-gamma-linolenic acid (DGLA)	$C_{20}H_{34}O_2$	[M+H]$^+$	307.2634	0.7	19.28	173.1318, 149.0950, 145.1005, 135.1119, 133.1019, 131.0827, 121.1034, 119.0868, 107.0860, 95.0855, 93.0722, 91.0548, 81.0717, 79.0578, 77.0419, 69.0722, 67.0570, 65.0421, 55.0561	↓*	↑#	↑#	HMDB 0002925	C03242
12	Eicosadienoic acid	$C_{20}H_{36}O_2$	[M+H]$^+$	309.2776	-3.6	19.73	161.1330, 147.1197, 137.0954, 135.1165, 133.1049, 121.1031, 119.0820, 111.1169, 109.1030, 107.0858, 97.1036, 95.0854, 93.0706, 91.0533, 83.0877, 81.0703, 79.0589, 69.0721, 67.0558, 57.0719, 55.0581	↓*	↑##	↑	HMDB 0005060 LMFA0 1030130	C16525
13	10Z-Nonadecenoic acid	$C_{19}H_{36}O_2$	[M+H]$^+$	297.2787	-0.2	21.30	191.1774, 177.1656, 163.1459, 149.1312, 139.1173, 135.1153, 125.0990, 123.1182, 121.1020, 111.0801, 109.1005, 107.0862, 97.1026, 95.0858, 93.0724, 91.0577, 85.1055, 83.0868, 81.0707, 79.0530, 77.0397, 71.0878, 69.0714, 67.0574, 57.0734, 55.0595	↓*	↑##	↑#	HMDB 0013622	/
14	Citric acid	$C_6H_8O_7$	[M-H]$^-$	191.0191	2.7	0.93	129.0165, 111.0080, 87.0087, 85.0285	↓*	↑##	↑###	HMDB 0000094	C00158
15	Glycerol-3-phosphate	$C_3H_9O_6P$	[M-H]$^-$	171.0063	-0.2	0.97	96.9721, 78.9597, 62.9710	↓***	↑	↑##	HMDB 0252849	C00093
16	N-Acetyl-D-tryptophan	$C_{13}H_{14}N_2O_3$	[M-H]$^-$	245.0909	1.3	5.00	142.0688, 118.0690, 115.0586, 91.0584	↑*	↓#	↓	HMDB 0255052	/

NO.	Metabolites	Formular	Ion mode	m/z	error	RT (min)	MS fragments (m/z)	MD	DZP	ZSSFH	HMDB ID	KEGG
17	Eicosapentaenoic acid (EPA)	$C_{20}H_{30}O_2$	$[M-H]^-$	301.2160	-1.4	9.22	283.2046, 257.2310, 229.1981, 203.1801, 175.1476, 135.1166, 71.0144, 59.0149	$\downarrow^{*}$	$\uparrow^{\#}$	$\uparrow^{\#\#\#\#}$	HMDB 0001999	C06428
18	Docosahexaenoic acid (DHA)	$C_{22}H_{32}O_2$	$[M-H]^-$	327.2308	-6.3	9.60	309.2246, 283.2421, 255.2133, 249.1972, 241.1927, 229.1954, 203.1815, 201.1670, 191.1843, 187.1511, 177.1642, 175.1489, 167.1099, 163.0756, 161.1351, 153.0928, 149.1359, 149.1359, 147.1213, 135.1193, 133.1026, 121.1066, 107.0873, 105.0743, 83.0523, 71.0151, 67.0559, 59.0154	$\downarrow^{**}$	$\uparrow^{\#\#}$	$\uparrow^{\#}$	HMDB 0002183	C06429
19	Arachidonic acid	$C_{20}H_{32}O_2$	$[M-H]^-$	303.2325	-1.4	9.67	301.2205, 285.2225, 259.2429, 231.2110, 205.1948, 177.0917, 59.0148	$\uparrow^{***}$	$\downarrow^{\#\#\#}$	$\downarrow^{\#\#\#}$	HMDB 0001043	C00219
20	Elaidic acid	$C_{18}H_{34}O_2$	$[M-H]^-$	281.2465	-7.4	10.25	263.2379, 127.0751, 71.0143	$\downarrow^{*}$	$\uparrow$	$\downarrow$	HMDB 0000573	C01712
21	14-Methylhexadecanoic Acid	$C_{17}H_{34}O_2$	$[M-H]^-$	269.2485	-0.1	10.55	251.2321	$\downarrow^{*}$	$\uparrow$	$\downarrow$	HMDB 0031067	/
22	Oleoyl-L-α-lysophosphatidic acid (LPA)	$C_{21}H_{41}O_7P$	$[M-H]^-$	435.2515	-1.2	12.18	417.2411, 281.2486, 152.9958, 96.9696	$\uparrow^{*}$	$\downarrow$	$\downarrow^{\#\#}$	HMDB 0242470	/

Note: ↑ means the abundance of metabolites was increased, while ↓ means the abundance of metabolites was decreased. * means MD group compared with CN group and # means DZP and ZSSFH groups compared with MD group. Data were expressed as mean ± SEM; n = 6. (*, # $p < 0.05$, **, ## $p < 0.01$, ***, ### $p < 0.001$)

Table S12. The number of r value > 0.9 in gut microbiota and SCFAs correlation analysis.

Phylum	SCFAs	Number (r > 0.9)
Firmicutes	Propionic acid	3
	Valeric acid	3
	Acetic acid	1
	Isobutyric acid	3
Bacteroidetes	Acetic acid	1
	Propionic acid	2
	Isobutyric acid	1
Proteobacteria	Valeric acid	1
	Isobutyric acid	1
	Acetic acid	1
Actinobacteria	Valeric acid	1
	Isobutyric acid	1
	Propionic acid	1

Table S13. R value > 0.9 in SCFAs and serum metabolites correlation analysis.

SCFAs	Serum metabolites	r
Acetic acid	Glycerol-3-phosphate	0.96
	Tryptophan	-0.91
	EPA	0.90
Propionic acid	LysoPC(18:1(9Z))	-0.91
	Glycerol-3-phosphate	0.92
	Tyrosine	-0.91
	Tryptophan	-0.92
Valeric acid	Glycerol-3-phosphate	0.94
	LysoPC(18:1(9Z))	-0.91
	Tyrosine	-0.97
	Tryptophan	-0.97
Isobutyric acid	Glycerol-3-phosphate	0.92
	LysoPC(18:1(9Z))	-0.91
	Tyrosine	-0.96
	Tryptophan	-0.95
